# Microstructures and Isothermal Oxidation of the Alumina Scale Forming Nb_1.7_Si_2.4_Ti_2.4_Al_3_Hf_0.5_ and Nb_1.3_Si_2.4_Ti_2.4_Al_3.5_Hf_0.4_ Alloys

**DOI:** 10.3390/ma12020222

**Published:** 2019-01-10

**Authors:** Mohammad Ghadyani, Claire Utton, Panos Tsakiropoulos

**Affiliations:** Department of Materials Science and Engineering, Sir Robert Hadfield Building, The University of Sheffield, Mappin Street, Sheffield S1 3JD, UK; m.ghadyani@sheffield.ac.uk (M.G.); c.utton@sheffield.ac.uk (C.U.)

**Keywords:** high entropy alloys, intermetallics, pest oxidation, high temperature oxidation, Nb–silicide based alloys, coatings, complex concentrated alloys, multi-principle element alloys

## Abstract

Nb–silicide based alloy will require some kind of coating system. Alumina forming alloys that are chemically compatible with the Nb–silicide based alloy substrate could be components of such systems. The intermetallic alloys Nb_1.7_Si_2.4_Ti_2.4_Al_3_Hf_0.5_ and Nb_1.3_Si_2.4_Ti_2.4_Al_3.5_Hf_0.4_ were studied in the cast, heat treated and isothermally oxidised conditions at 800 and 1200 °C to find out if they are alumina scale formers. The alloys were designed using the alloy design methodology NICE and were required (i) not to have stable solid solution phase in their microstructures; (ii) not to pest and (iii) to form alumina scale. Their microstructures consisted of silicides and aluminides. Both alloys satisfied (i) and (ii) and formed thin scales at 800 °C. At 1200 °C the former alloy suffered from internal oxidation and formed alumina intermixed with Ti rich oxide beneath a thick “layered” scale of mixed oxides that contained Ti and/or Al and/or Si. There was no internal oxidation in the latter alloy that formed a thin continuous well adhering α-Al_2_O_3_ scale that was able to repair itself during oxidation at 1200 °C. In both alloys there was severe macrosegregation of Si, which in Nb_1.3_Si_2.4_Ti_2.4_Al_3.5_Hf_0.4_ was almost double that in Nb_1.7_Si_2.4_Ti_2.4_Al_3_Hf_0.5_. The severe macrosegregation of Si contributed to the formation of a “layered” structure in the former alloy that was retained at 800 and 1200 °C. Both alloys met the “standard definition” of High Entropy Alloys (HEAs). Compared with the range of values of the parameters valence band (VEC), δ and Δχ of bcc solid solution plus intermetallic(s) HEAs, only the Δχ of the alloy Nb_1.7_Si_2.4_Ti_2.4_Al_3_Hf_0.5_ was within the range and the parameters VEC and δ of both alloys respectively were outside and within the corresponding ranges. The alloy Nb_1.3_Si_2.4_Ti_2.4_Al_3.5_Hf_0.4_ exhibited strong correlations between the parameters Δχ, δ and VEC, and the range of values of each parameter was wider compared with the alloy Nb_1.7_Si_2.4_Ti_2.4_Al_3_Hf_0.5_. There was a strong correlation only between the parameters Δχ and δ of the latter alloy that was similar to that of the former alloy.

## 1. Introduction

Nb-silicide based alloys could replace Ni-based superalloys in advanced gas turbines to enable the latter to operate at higher turbine entry temperatures so that engine performance targets with new and stringent environmental targets can be met. These alloys have microstructures that contain bcc Nb solid solution (Nb_ss_), tetragonal and/or hexagonal Nb_5_Si_3_ silicides and other intermetallic compounds, such as tetragonal Nb_3_Si silicide, C14-NbCr_2_ Laves phase and A15–Nb_3_X (X = Al, Ge, Si, Sn) compounds. The Nb_ss_ and the intermetallic compounds are alloyed [1,2,3]. For example, the Nb_5_Si_3_ can be very rich in Ti and Hf. The volume fractions of Nb_ss_ and Nb_5_Si_3_ are important for achieving a balance of creep, oxidation and toughness properties. A high vol.% of Nb_ss_ is disadvantageous to creep and oxidation.

A strategic objective of the development of Nb–silicide based alloys is the improvement of their oxidation. The approach used to achieve the latter has been to find out which alloying additions affect oxidation. Research has demonstrated that the alloying elements Al, B, Cr, Fe, Ge, Hf, Sn and Ti improve the oxidation of Nb–Si based alloys. Alloy development has shown that Nb–silicide based alloys can offer a balance of properties.

Oxidation resistance often controls the life of high temperature alloys in structural engineering applications. Dense, continuous and adherent Al_2_O_3_ or SiO_2_ oxides protect alloys from oxidation at high temperatures (*T* > 1000 °C). These oxides are the most protective, because of their high thermodynamic stability and the low diffusivities for anions and cations. Unfortunately, Nb–silicide based alloys are not alumina formers, because in these alloys the concentration of Al must be kept low owing to the adverse effect of this element on mechanical properties (ductile to brittle transition temperature-DBTT of the Nb_ss_, toughness, high temperature strength and creep of the alloy). Furthermore, the concentration of Si in these alloys, which can be as high as 20 at.%, cannot assure the formation of silica scale. In other words, the Nb–silicide based alloys are not alumina or silica formers. Instead, their scales consist of Ti niobates, AlNbO_4_, CrNbO_4_ and oxides of Nb and Ti [4,5].

In the operating environment of an aero-engine, the Nb–silicide based alloys will require protection via some kind of coating system. A requirement of the coating system is chemical compatibility with the substrate. One approach to the design of a coating system for Nb–silicide based alloys is to consider thermal barrier type coating systems consisting of a bond coat and top coat, where the bond coat could be a layered multi-material system or a functionally graded material forming in situ αAl_2_O_3_ between the bond coat and the top coat. A layered multi-material coating system has been suggested by Jackson et al. [6].

Is there αAl_2_O_3_ forming alloy(s) that could be used in coating system(s) compatible with Nb–silicide based alloys? The need to answer this question has motivated investigations in our research group that resulted in the research presented in this paper. The latter focusses on two Nb–Ti–Si–Al–Hf alloys that were studied as part of an ongoing research programme that aims to discover which (if any) alloys of Al–Hf–Nb–Si–Ti–X systems are alumina formers. The two alloys were not studied as coatings applied on a Nb–silicide based substrate in order to eliminate the effects of substrate and coating process on microstructure and oxidation.

The structure of the paper is as follows. First the approach used to design and select the two alloys is explained. Then the experimental techniques used for the characterisation of the alloys are described. The results for the cast and heat treated alloys are presented before their isothermal oxidation at 800 and 1200 °C is presented. The discussion considers first the macrosegregation and microstructures of the alloys, which are also compared with High Entropy Alloys, and then their oxidation behaviour is considered.

## 2. Design and Selection of the Alloys of This Research

Our goal was to design and develop αAl_2_O_3_ scale forming Nb–Ti–Si–Al–Hf alloys. The design of the alloys studied in the research reported in this paper was guided by the alloy design methodology NICE, which was recently described in Reference [4], and current knowledge about the oxidation of Nb–Si based alloys. Briefly, in NICE there are three key parameters that guide the design (selection) of Nb–Si based alloys. These are based on electronegativity (Δχ), atomic size (δ) and number of valence electrons per atom filled into the valence band (VEC). There are relationships between these parameters and the concentrations of elements in alloys and the weight gains of the latter in isothermal oxidation. These relationships were discussed in Reference [4].

The Nb_ss_ is known to be the Achilles’ heel in the oxidation of Nb–Si based alloys. We decided to design alloys (i) with zero volume fraction of Nb_ss_ and (ii) with microstructures that should contain Al rich, Si rich and/or Al and Si rich intermetallic phases, in particular transition metal aluminides and silicides. The choice of intermetallics was guided by the literature [7,8,9,10,11,12,13,14,15,16,17,18,19,20,21,22,23,24]. Below we discuss why we preferred certain intermetallic compounds to be stable in the microstructures of our alloys.

### 2.1. Which Intermetallic Compounds?

We did not want the alloys to exhibit catastrophic pest oxidation. Taking into consideration the alloying elements that are known to improve the oxidation of Nb–silicide based alloys (see Section 1) a good starting point was to consider the Ti–Al system. The intermetallic compounds of the Ti–Al system are known not to pest [7]. Aluminium rich TiAl, and the TiAl_2_ and TiAl_3_ aluminides can form alumina scales but Al poor TiAl and Ti_3_Al form titania rich scales and oxidise at much higher rates [8]. For example, when TiAl with 50 at.% Al was oxidised in air at 950, 1100 and 1200 °C, at the two higher temperatures TiO_2_ scale formed and there was internal oxidation. Parabolic oxidation kinetics were followed at the low temperature where the oxidation was independent of specimen preparation and fabrication method [9]. Furthermore, the alloying of TiAl with Nb promoted the formation of continuous alumina scale on TiAl (50 at.% Al) at 950 °C independent of surface preparation or exposure environment (air or O_2_) but the alloying with Hf had a minimal effect [9]. The alloying of TiAl with Si improved oxidation resistance [10]. For Al poor TiAl it is known that the addition of 4 to 12 at.% Nb in Ti–48Al increased oxidation resistance at 850 °C and that the alloys developed continuous bands of dense alumina beneath titania rich surface layer that formed during the early stages of oxidation [11]. When TiAl_2_, which was sputter deposited on TiAl, was oxidised in air at 800 and 900 °C, only external well adhering almost micro-crack free θ-Al_2_O_3_ scale was formed on the TiAl_2_ [12]. In the isothermal oxidation of arc melted TiAl_3_ parabolic oxidation kinetics were followed above 1000 °C for 100 h in flowing oxygen and αAl_2_O_3_ scale formed. Cyclic oxidation tests at 982 °C also confirmed that αAl_2_O_3_ formed after 20 cycles [13].

With Nb and Al as constituent elements of the alloys to be designed the oxidation of intermetallic compounds in the Nb–Al binary system also was considered. NbAl_3_, which is isomorphous with TiAl_3_, is the only compound in the Nb–Al system that can form continuous αAl_2_O_3_ scale at high temperatures [14] but it is known to pest, with the worst behaviour exhibited between 650 and 850 °C [15]. Unlike TiAl_3_, the NbAl_3_ aluminide has a narrow solubility range. Like the TiAl_3_, the depletion of Al by the initial formation of the oxide layer results in the formation of a lower compound, namely Nb_2_Al, beneath the scale. The latter compound influences the structure, stability and adherence of the oxide layer. Nb_2_Al cannot form continuous alumina. Rupture of the initial alumina layer is followed by the rapid growth of AlNbO_4_ and Nb_2_O_5_ and the consumption of Nb_2_Al, which is then followed by the growth of alumina again on the NbAl_3_. Repetition of this process results in a layered scale and nearly linear oxidation kinetics. Excess Al prevents the formation of the layered structure but degrades the long term oxidation resistance, because of Al evaporation and alumina growth in grain boundaries. However, alloying the NbAl_3_ with Ti promotes external αAl_2_O_3_ scale formation at lower Al concentrations than those required for the binary alloys [7].

Taking into account that Ti and Si are key alloying elements that are known to improve the oxidation of Nb–silicide based alloys (see Section 1) we also considered the Ti–Si system. The intermetallic compounds of the Ti–Si system do not suffer from pest oxidation [15]. We were not interested in the Ti_3_Si compound, because it is isomorphous with Nb_3_Si and the latter is known to pest. However, we were interested in the Ti_5_Si_3_, because it is isomorphous with the hexagonal Nb_5_Si_3_ and for the following reasons. The Ti_5_Si_3_ has excellent oxidation resistance in oxygen at *T* > 1000 °C but insufficient oxidation resistance in air [16]. Also, it has excellent oxidation resistance in air at 1200 °C when it contains small vol.% of Ti_5_Si_4_ or TiSi_2_ [17,18]. For example, arc melted Ti_5_Si_3_ gained weight 31 mg/cm^2^ after cyclic oxidation at 1149 °C for 50 h and its scale consisted of TiO_2_ (about 80 vol.%) and SiO_2_ (α-cristobalite) [19] but for Si-depleted Ti_5_Si_2.8_ the initial formation of SiO_2_ was not favoured and TiO_2_ grew on the surface, while for the Si-rich Ti_5_Si_3.2_ the SiO_2_ was more favourable than TiO_2_ [18].

With Nb and Si in the alloys to be designed we also considered the oxidation of intermetallic phases in the Nb–Si system. The Nb_3_Si, Nb_5_Si_3_ and NbSi_2_ compounds pest in the temperature range 700 to 850 °C forming Nb_2_O_5_. In Nb–silicide based alloys, Nb_5_Si_3_ silicide grains can be contaminated by oxygen [20]. Interstitials can stabilise the hexagonal γNb_5_Si_3_ (hP16, D8_8_, prototype Mn_5_Si_3_) [21]. The hexagonal Ti_5_Si_3_, owing to its Mn_5_Si_3_-type structure (isomorphous with γNb_5_Si_3_), can incorporate interstitial ternary additions (has one interstitial site per formula unit corresponding to about 10 at.% at 1000 °C [22]) that modify its oxidation resistance without changing the crystal structure [19]. The concentration of interstitial oxygen in Ti_5_Si_3_ is about 6 at.% [18]. Interstitial ternary oxygen additions increased the oxidation resistance of binary Ti_5_Si_3_, for example the weight gain of Ti_5_Si_3_O_0.25_ at 1000 °C was 0.45 mg/cm^2^ after 240 h, and the weight gain of Ti_5_Si_3_O_0.75_ at 1079 °C was 0.82 mg/cm^2^ after 130 h while at 1306 °C the weight gain was 1.1 mg/cm^2^ after 240 h [19]. The scale formed on Ti_5_Si_3_ doped with interstitial oxygen consisted of crystalline silica matrix forming a continuous layer that contained titania particles. The alloying with oxygen promoted the formation of thin silica layer in the early stages of oxidation [18].

The microstructure of Ti_5_Si_3_-8 wt.% Al contained a dispersion of TiAl_3_ (Ti_0.25_(Al_0.67_Si_0.08_)) of about 15 vol.% and Al_2_O_3_ and a small volume fraction of Ti_5_Si_4_ in which the concentration of Al was very low [17]. The scale formed on the Ti_5_Si_3_-8 wt.% Al alloy after 80 h at 1200 °C in air was about 30 μm thick and contained αAl_2_O_3_ and TiO_2_ and no SiO_2_ [17]. The scale was made of two layers; an outer overlapping layer of Al_2_O_3_ and TiO_2_ and an inner layer of Al_2_O_3_ [17]. There was a depletion of the TiAl_3_ compound and an increase of the vol.% of Al_2_O_3_ in the substrate after the oxidation [17]. It is also known that the alloying of Ti_5_Si_3_ with Nb within the solubility limit (about 15.6 at.%) improved the oxidation resistance in flowing dry air at 900 °C of single crystal alloys that were produced using the Czochralski method [23]. Finally, it has been reported that a Ti(Al*_x_*Si_1−*x*_)_2_ (0.15 < *x* < 0.3) coating on Ti–6Al–4V substrate decomposed to a layered structure that consisted of the Ti_5_Si_4_ and TiSi silicides at 850 and 950 °C and that the latter significantly improved the oxidation resistance [24].

In summary, (a) Al rich TiAl, TiAl_2_ and TiAl_3_ can form αAl_2_O_3_; (b) Nb and Si benefit the oxidation of TiAl; (c) Ti improves the oxidation of NbAl_3_ that can form αAl_2_O_3_; (d) Ti_5_Si_3_ has oxidation resistance at 1200 °C when in synergy with (in the presence of) small vol.% of Ti_5_Si_4_ and TiAl_3_; (e) interstitial oxygen in Ti_5_Si_3_ improves its oxidation resistance; and (f) alloying Ti_5_Si_3_ with Al suppresses SiO_2_ formation, and promotes formation of αAl_2_O_3_ beneath alumina and titania scale in air at 1200 °C.

Therefore, the literature guided us to aim to have in the microstructures of the alloys to be designed (a) Al rich TiAl, TiAl_2_ and TiAl_3_ aluminides where Ti would be substituted by Nb and Hf, and Al by Si and (b) Me_5_Si_3_ and Me_5_Si_4_ silicides, where Me is transition metal; and (c) to avoid the formation of Nb rich tri-aluminide. In particular, Me_5_Si_3_ silicide of hexagonal structure was desirable. We had good reasons to believe that the latter was possible in alloys of our chosen system (see Section 1), because our previous research had indicated that hexagonal Nb_5_Si_3_ would be the stable silicide in the Nb–24Ti–18Si–5Al–5Hf alloy [20].

### 2.2. Alloy Design

When we considered the requirement for zero volume fraction of Nb_ss_ (see (i) in the previous section), the alloy design methodology NICE gave the following values for the parameters Δχ, VEC and δ: Δχ = 0.1543, VEC = 4.263 and δ = 9.0075. For these values the NICE gives the following concentrations: Ti = 24.2 at.%, Si = 21.5 at.% and Hf = 4.7 at.%. Next, we calculated the concentrations of these elements for the extreme (ideal) condition of Δ*W*/*A* (weight gain per unit area) equal to zero at 800 and 1200 °C, for which the NICE gave the following concentrations: Ti = 21.3 at.%, Si = 22.3 at.% and Hf = 4.4 at.%.

In view of the high but non-dissimilar concentrations of Si and Ti calculated from the two approaches based on NICE, we considered if Me_5_Si_3_ silicides could be in equilibrium with Al-rich aluminides. The available Ti–Al–Si phase equilibria data [25] shows that Ti_5_Si_3_ can be in equilibrium with TiAl and TiAl_2_ for Al ≈ 24 at.% and Si ≈ 24 at.% or in equilibrium with TiAl_3_ and/or TiAl_2_ for Al ≈ 30 at.% and Si ≈ 24 at.% or in equilibrium with TiAl_3_ and Ti_5_Si_4_ for Al ≈ 35 at.% and Si ≈ 24 at.%.

The literature on the oxidation of Ti–Al alloys shows that protective Al_2_O_3_ scale does not necessarily form when alumina is thermodynamically stable in the alloy and that higher Al concentrations are required for kinetic reasons [26]. In other words, there are “two” Al concentrations, one required to thermodynamically stabilise Al_2_O_3_, we shall call this *C*_ther_^Al^, and the other, which we shall call *C*_kin_^Al^ (*C*_kin_^Al^ ≥ *C*_ther_^Al^), required to form a continuous protective oxide. Thermodynamics dominate alumina formation when *C*_kin_^Al^ = *C*_ther_^Al^ and kinetics when *C*_kin_^Al^ > *C*_ther_^Al^. The same is the case for Ti–Si alloys where the minimum Si concentration to form SiO_2_ is about 40 to 45 at.% [26]. Alloying additions may change *C*_ther_^Al^, the oxygen solubility and the diffusivities of oxygen and Al in the alloy and thus may affect the concentration of Al at the oxide/alloy interface (see discussion). Kinetic factors would reduce *C*_kin_^Al^ − *C*_ther_^Al^ (for example, see in previous section comment about the addition of Nb in Ti–48Al).

The calculated concentrations of Hf, Si and Ti from NICE, and the Al concentration for which Me_5_Si_3_ silicide is in equilibrium with TiAl and TiAl_2_ (i.e., thermodynamics) were “guiding us” to consider an alloy of composition 23.75Nb–23.75Si–23.75Ti–23.75Al–5Hf (at.%). This could be considered to be a “High Entropy Alloy” (HEA) or a “Multi-Principle Element Alloy” (MPEA), or a “Complex Concentrated Alloy” (CCA). Then again, the oxidation literature was “guiding” us to increase the Al concentration.

We know that Nb–silicide based alloys with high vol.% Nb_ss_ exhibit lower Si macrosegregation compared with alloys with low vol.% Nb_ss_ for the reasons discussed in Reference [27]. For the (still to be decided) intermetallic alloys to be studied in this paper, because of the requirement for zero vol.% Nb_ss_ (see (i) at the beginning of this section), we would expect the Si macrosegregation to be high. When high Si macrosegregation was observed in Nb–silicide based alloys with high vol.% of intermetallics, the alloys had higher Δ*H*_m_/*T*_m_ and *T*_m_^sp^ values and lower *T*_m_ and *T*_m_^sd^/*T*_m_^sp^ values compared with the alloys with low Si macrosegregation (see Reference [27] for the definition of and equations for the parameters used for the study of macrosegregation). The low *T*_m_ accounts for the formation of undercooled melt near an effective heat sink, such as the walls of the water cooled copper crucibles used for the preparation of alloys. High melt undercooling is required for the growth of faceted *S*/*L* interfaces, like those of intermetallic compounds that have high entropy of fusion. High Δ*H*_m_/*T*_m_ is consistent with high vol.% of intermetallic compounds in an alloy [27]. Zone(s) with different microstructure(s) can form from undercooled melts of Nb–silicide based alloys, for examples see References [28,29,30,31]. The high *T*_m_^sp^ and low *T*_m_ and *T*_m_^sd^/*T*_m_^sp^ values steered us to high concentration of Al. Taking into consideration that Al and Ti in Nb alloy melts are “surface active” elements that tend to segregate to the surface [32], an increase in Al concentration with the accompanied changes in the aforementioned parameters that describe macrosegregation could lead to different zones forming in the alloy(s) from the bottom of the buttons (in contact with the heat sink) towards the bulk. In other words, the increase of the Al concentration and the solidification conditions at an effective heat sink could possibly result in some form of “functionally gradient microstructure”.

Next, we decided to select the following Si, Ti and Hf concentrations, Si = 23.75 at.%, Ti = 23.75 at.% and Hf = 5 at.%, and to opt for Al = 30 at.%, anticipating to avoid having stable TiAl in the microstructure. This approach gave the Nb concentration (balance) of 17.5 at.%. Thus, the nominal composition of our first alloy was 17Nb–24Si–24Ti–30Al–5Hf or Nb_1.7_Si_2.4_Ti_2.4_Al_3_Hf_0.5_. Subsequently, we decided to increase the volume fraction of tri-aluminide in the microstructure and also to exploit the presence of Ti_5_Si_4_ in it (see above in this section). This required us to increase the concentration of Al. We opted for Al = 35 at.% and Hf = 4 at.%. Thus, the nominal composition of our second alloy was 13Nb–24Si–24Ti–35Al–4Hf or Nb_1.3_Si_2.4_Ti_2.4_Al_3.5_Hf_0.4_. Both alloys may be considered to be High Entropy Alloys (HEAs) or Multi-Principle Element Alloys (MPEAs) or Complex Concentrated Alloys (CCAs). For the aforementioned alloys we could not calculate the Al concentrations that correspond to *C*_ther_^Al^ or *C*_kin_^Al^ (see Section 5.3).

In summary, guided by the literature on the oxidation of intermetallic compounds, the alloy design methodology NICE, the available phase equilibria data and data about macrosegregation in Nb-Si based alloys, we selected two alloys, namely the alloys Nb_1.7_Si_2.4_Ti_2.4_Al_3_Hf_0.5_ and Nb_1.3_Si_2.4_Ti_2.4_Al_3.5_Hf_0.4_, which we wanted to have (i) zero vol.% Nb_ss_ and (ii) microstructures consisting of Me*_x_*Al*_y_* aluminides and Me*_x_*Si*_y_* silicides, and form alumina scales at 800 and 1200 °C. Also, we were interested to find out if zones of different microstructures would form in these alloys.

## 3. Experimental

Buttons (25 g) of the two alloys were prepared from high purity (better than 99.9 wt.%) elements by arc-melting in an argon atmosphere using a non-consumable tungsten electrode in a water cooled copper crucible. The melting procedure was repeated 5 times for each alloy. The samples for heat treatment were wrapped in Ta foil and placed in an alumina boat in the hot zone of a tube furnace. A crucible containing Ti-sponge was placed in the entrance of the tube furnace to ensure that the heat treatments were carried out in flowing Ti gettered argon. The alloy Nb_1.7_Si_2.4_Ti_2.4_Al_3_Hf_0.5_ was heat treated at 1300 °C and the alloy Nb_1.3_Si_2.4_Ti_2.4_Al_3.5_Hf_0.4_ at 800 and 1200 °C. The latter temperatures were the same as those used for the isothermal oxidation experiments.

Cube specimens (approximately 0.4 cm × 0.4 cm × 0.4 cm) cut from the as cast buttons were prepared for isothermal oxidation. The specimens were polished to 300 grit. Isothermal oxidation experiments were performed at 800 and 1200 °C for 100 h using a NETZSCH STA 49 F3 Jupiter thermal analyser (NETZSCH Gmbh, Selb, Germany) supported by the NETZSCH Proteus software. The instrument had a weight resolution of 0.1 µg over the entire weighing range (0–35,000 mg). We used a 3 degrees per minute heating rate from room temperature to 800 or 1200 °C. A Jeol 6400 scanning electron microscope (SEM, Jeol, Tokyo, Japan) and a Philips XL 30S FEG SEM (Philips-ThermoFisher Scientific, Hillsboro, OR, USA) were used for imaging and quantitative analysis. Both instruments were equipped with EDS detectors and Oxford Instrumentals INCA software for quantitative chemical analysis, and elemental standards of Nb, Ti, Al, Si, Hf. The Philips XL 30S FEG SEM was also equipped with Fe_2_O_3_ as the standard for oxygen. The X-ray maps of scales were taken in the latter instrument. All compositions in this paper are given in at.% unless stated differently.

A Siemens D5000 diffractometer with a Cu Kα (Hiltonbrooks Ltd, Crew, UK) was used for phase identification in the as cast and heat treated specimen. The same diffractometer was used for glancing angle XRD to identify the oxides in the scales that formed on the oxidised specimens. The glancing angle XRD was performed at a scan speed of 2°/min over a 2θ range of 20° to 100° with a glancing angle of 5°. For phase analysis the ICDD (International Centre for Diffraction Data) PDF-4+ database and Sieve+ software (ICDD, Newtown Square, PA, USA) was used.

## 4. Results

### 4.1. Alloy Nb_1.7_Si_2.4_Ti_2.4_Al_3_Hf_0.5_

**Figure 1:**
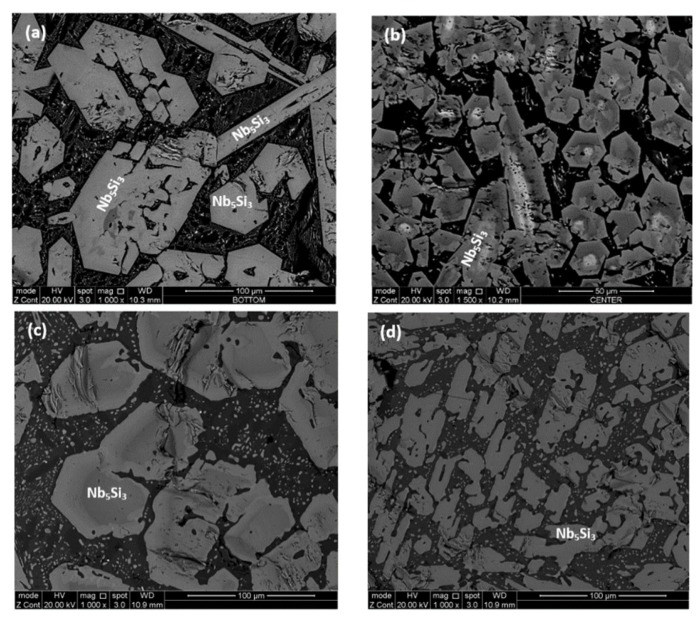
Scanning electron microscope (SEM) backscatter electron images of the alloy Nb_1.7_Si_2.4_Ti_2.4_Al_3_Hf_0.5_, (**a**,**b**) cast alloy; (**c**,**d**) heat treated alloy (1300 °C/100 h). (**a**,**c**) top; (**b**,**d**) bulk.

As cast: The actual composition of the alloy was Nb–23.4Ti–22.8Si–29.7Al–4.8Hf. This was the average composition of all EDS analyses taken from the top, bulk and bottom of the button. The standard deviations of the concentrations of all the elements with the exception of Hf were greater than one, indicating chemical inhomogeneity in the microstructure (see below). The cast microstructure is shown in Figure 1a,b. In all parts of the button there were large (bulky) faceted grains of a light contrast phase surrounded by a darker contrast microstructure, in which there were fine second phase(s) that were not easy to distinguish owing to similarities in contrast. The vol.% of the darker contrast microstructure was significantly reduced in the bottom of the button.

**Figure 2:**
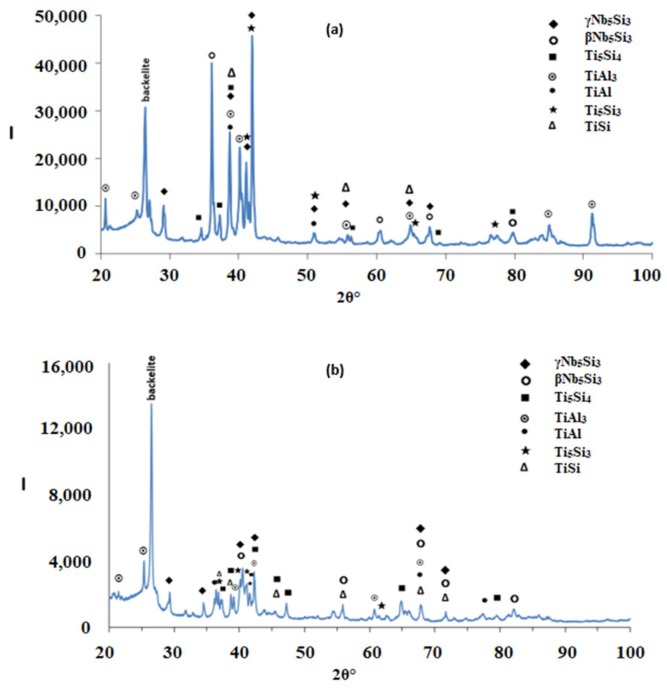
X-ray diffractograms of the alloy Nb_1.7_Si_2.4_Ti_2.4_Al_3_Hf_0.5_, (**a**) as cast; (**b**) heat treated (1300 °C/100 h).

According to the XRD data (Figure 2a), silicides and TiAl*_x_* (*x* = 1, 3) aluminides were present in the microstructure. In the X-ray diffractogram there were peaks that corresponded only to hexagonal γNb_5_Si_3_, or tetragonal βNb_5_Si_3_ or TiAl_3_, and the peaks for TiAl coincided with those of other phases. The outline of some of the large lighter contrast grains in Figure 1a,b suggested hexagonal symmetry, which is consistent with the crystal structure of γNb_5_Si_3_. The XRD data also suggested the presence of Ti_5_Si_4_ and TiSi. Peaks of the latter silicide coincided with peaks of other phases. The Ti_5_Si_4_ forms as thin layers on Ti_5_Si_3_ and the TiSi has similar contrast with Ti_5_Si_4_ (see Section 4.2 and discussion). Careful study of the cast alloy using EDS did not confirm the existence of Ti_5_Si_4_ and TiSi.

**Figure 3:**
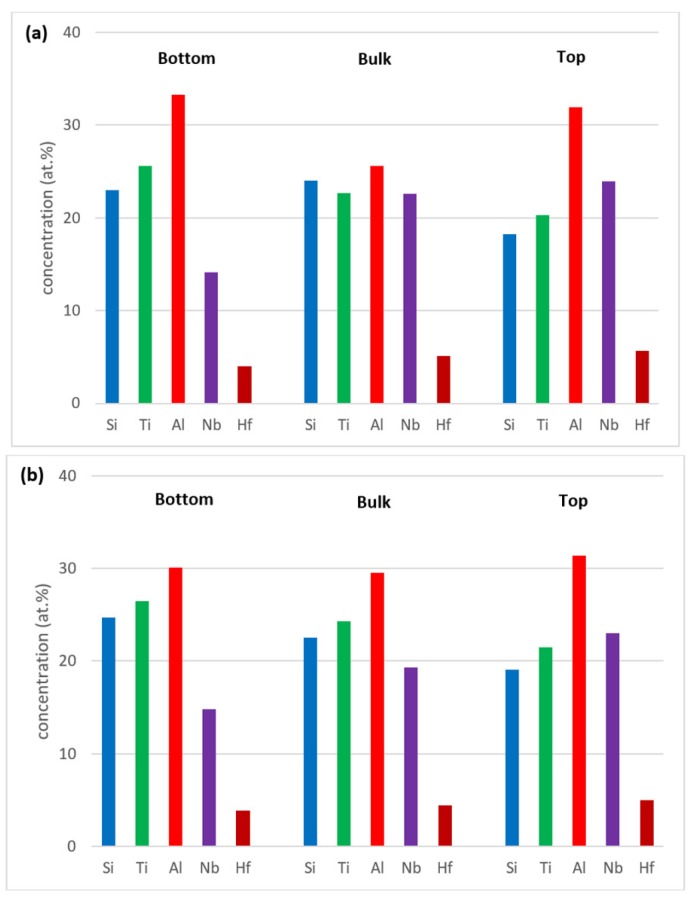
Comparison of the concentrations of elements in the bottom, bulk and top of the alloy Nb_1.7_Si_2.4_Ti_2.4_Al_3_Hf_0.5_ (**a**) as cast; (**b**) heat treated at 1300 °C.

Zones of different microstructure were not observed in the cross sections of the cast alloy even though there were differences in composition between bottom, bulk and top of the button, see Figure 3a. The bulk was poorer in Al compared with the bottom and top, the bottom was richer in Ti than the bulk and top and the top was poorer in Si than the bottom and bulk.

The EDS analysis confirmed that the large lighter contrast grains were Nb_5_Si_3_ with average composition 43.1Nb–31.2Si–12.8Ti–7Al–5.7Hf. There was segregation of Ti and Hf in the Nb_5_Si_3_, some grains were Ti-rich with average composition 21.9Nb–38Si–31.2Ti–3Al–5.8Hf and others Hf rich with average composition 32.4Nb–35.9Si–18.5Ti–4.8Al–8.4Hf. Thus, owing to the partitioning of Ti and Hf some silicide grains had Nb/(Ti + Hf) ratio significantly less than 1 and others approximately equal to one or higher than one, which would indicate the presence of γNb_5_Si_3_ and tetragonal Nb_5_Si_3_ respectively [33]. This is consistent with the morphology of the silicides in Figure 1a,b, which shows hexagonal symmetry, and with the XRD data.

The microstructures surrounding the Nb_5_Si_3_ grains exhibited dark contrast but within these dark contrast areas there was also a slight variation in contrast which is not easy to reproduce in Figure 1. The EDS analyses confirmed different Al + Si concentration in different phases in these areas some of which were Ti rich, and others Ti and Nb rich. With the guidance of the XRD data (Figure 2a) and the works of Dezellus et al. [34], Bulanova et al. [35], Perrot [36] and Park et al. [37,38], these phases were identified to be aluminides, in particular TiAl_3_ and (Ti,Nb)Al_3_, TiAl, Ti_2_Al_5_, and the compounds TM_2.35_Al_1.65_Si and TM_3.7_Al_3_Si. The average compositions of these phases were as follows: TiAl_3_ = 1.4Nb–2.7Si–22Ti–71.9Al–1.4Hf, (Ti,Nb)Al_3_ = 6.5Nb–2.1Si–17.5Ti–72.1Al–1.6Hf, TiAl = 16.2Nb–0.9SSi–20.3Ti–58.2Al–4.2Hf and 18.8Nb–3.5Si–19.7Ti–53.3Al–4.6Hf, Ti_2_Al_5_ = 16Nb–1.1Si–10.8Ti–70.8Al–1.4Hf, TM_2.35_Al_1.65_Si = 13Nb–19.8Si–26.5Ti–33.3Al–7.1Hf, and TM_3.7_Al_3_Si = 18.7Nb–12.9Si–20.4Ti–39.2Al–8.8Hf. The heat treatment confirmed that the latter two compounds were metastable phases that formed because of the strong chemical inhomogeneity that existed in the cast alloy. Finally, in the top of the button some of the microstructure in the dark contrast area was similar to that of a eutectic with average composition 19.8Nb–6.1Si–20.2Ti–49.4Al–4.5Hf. The Si and Al concentrations of the latter were in agreement with that of a eutectic between Nb_5_Si_3_ and TiAl reported in References [35,36].

In summary, different intermetallic compounds were present in different parts of the as cast button. The Hf rich Nb_5_Si_3_ and tetragonal Nb_5_Si_3_, TiAl and TM_3.7_Al_3_Si were observed in the top. In the bottom we found only TiAl_3_ and (Ti,Nb)Al_3_ and the Ti rich Nb_5_Si_3_ and in the bulk we observed the Ti and Hf rich Nb_5_Si_3_ and TiAl, Ti_2_Al_5_ and Ti_2.35_Al_1.65_Si.

Heat treated: The average composition of the heat treated alloy (1300 °C/100 h) was 18.8Nb–22.3Si–24.3Ti–30.2Al–4.4Hf, and was close to that of the cast alloy. The standard deviations of the concentrations of all the elements with the exception of Hf were still high owing to the prevailing large scale chemical inhomogeneity after the heat treatment. The microstructure is shown in Figure 1c,d, and the XRD data in Figure 2b. After the heat treatment, zone(s) were not observed, and the average composition of the bottom, bulk and top areas had not changed significantly (Figure 3b).

The microstructure consisted of the Nb_5_Si_3_, (Ti,Nb)Al_3_, TiAl and Ti_2_Al_5_ intermetallics and there was still segregation of Ti and Hf in the silicide. The average compositions were as follows: Nb_5_Si_3_ = 29.9Nb–36.6Si–22.1Ti–4Al–7.4Hf and 20.7Nb–36.6Si–35.1Ti–2.6Al–5.1Hf, (Ti,Nb)Al_3_ = 17.8Nb–9.6Ti–71.7Al–1Hf, TiAl = 18.7Nb–1.8Si–20.8Ti–56.3Al–2.3Hf and Ti_2_Al_5_ = 20.7Nb–0.6Si–7.3Ti–70.2Al–1Hf. There were Nb_5_Si_3_ grains with Nb/(Ti + Hf) ≈ 0.5, which would correspond to hexagonal Nb_5_Si_3_ [33]. The TM_2.35_Al_1.65_Si and TM_3.7_Al_3_Si phases were not observed.

The XRD data suggested the presence of TiAl and TMAl_3_, and tetragonal βNb_5_Si_3_ and hexagonal γNb_5_Si_3_. However, the EDS data and the Nb/(Ti + Hf) ratio of the 5-3 silicide grains, which was reduced to less than 1 after the heat treatment, would suggest that the hexagonal γNb_5_Si_3_ is most likely the stable silicide in the microstructure of this alloy. The stable aluminides in this alloy were the TiAl, (Ti,Nb) Al_3_ and possibly the Ti_2_Al_5_. There was no evidence of the prior eutectic but the microstructure that surrounded the bulky Nb_5_Si_3_ grains exhibited light contrast particles in the dark contrast matrix, which is consistent with coarsened Nb_5_Si_3_ + TiAl prior eutectic. The XRD also indicated the presence of the Ti_5_Si_4_ and TiSi silicides, which were not confirmed by EDS analysis.

### 4.2. Alloy Nb_1.3_Si_2.4_Ti_2.4_Al_3.5_Hf_0.4_

**Figure 4:**
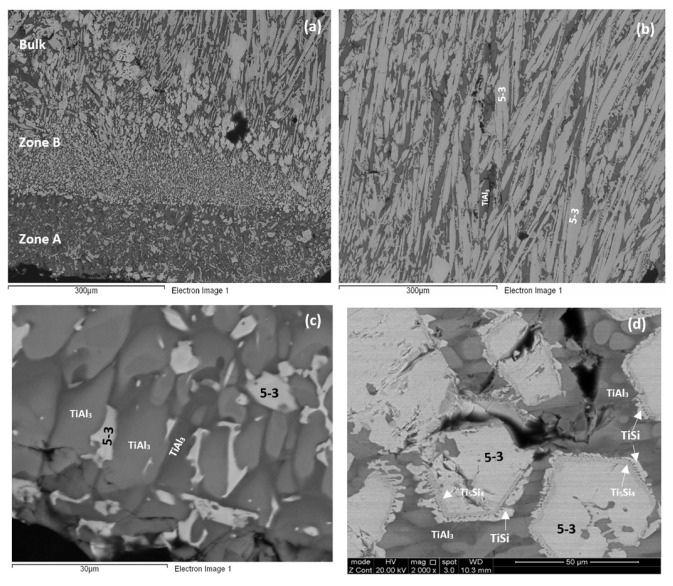
SEM backscatter electron images of the cast alloy Nb_1.3_Si_2.4_Ti_2.4_Al_3.5_Hf_0.4_. (**a**,**b**) low magnification images (**a**) showing zones A, B and transition to bulk microstructure; (**b**) bulk microstructure; (**c**,**d**) higher magnification images showing (**c**) details of microstructure in Zone A and (**d**) bulk microstructure.

As cast: The cast microstructure demonstrated the sensitivity of the solidification of this alloy to high temperature gradient(s) and/or cooling rate(s). Indeed, cross sections exhibited a “layered” structure separating the bottom from the bulk. The former consisted of two zones, namely Zone A and Zone B. There were differences in the contrasts of the zones A and B and the bulk and top of the cross section owing to differences in zone thickness, the scale and morphology of the microstructure, the chemical inhomogeneity and transitions in microstructure, see Figure 4a. Next to the water cooled copper crucible (highest temperature gradient(s) and/or cooling rate(s)) Zone A formed that had more or less a constant thickness. Then was Zone B the thickness of which varied slightly as the microstructure changed to that observed in the bulk.

The actual composition of the alloy was Nb–23.8Si–23.5Ti–35.9Al–3.3Hf. This was the average composition of all EDS analyses taken from the top, bulk and bottom of the button and was very close to the nominal one. However, the standard deviations of the concentrations of all the elements with the exception of Hf were greater than one, particularly those of Al and Si, owing to the changes (transitions) in microstructure and differences in the compositions of phases (see below). For the bulk and top of the button the standard deviations of all elements were smaller but still larger than one, and less and/or equal to one only for Hf and Ti.

**Figure 5:**
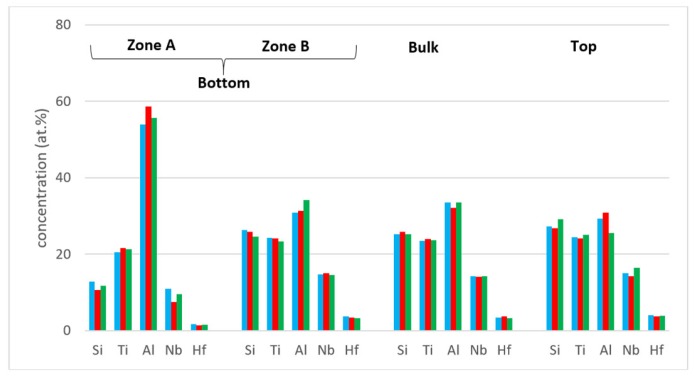
Comparison of the concentrations of elements in the bottom, bulk and top of the alloy Nb_1.3_Si_2.4_Ti_2.4_Al_3.5_Hf_0.4_. As cast-blue bars, heat treated at 800 °C—red bars and heat treated at 1200 °C—green bars.

The concentrations of each element in the two zones in the bottom, bulk and top of the cast alloy are shown by the blue colour vertical bars in Figure 5. The latter shows that Zone A was richer in Al and poorer in Hf, Nb, Si and Ti than the rest of the alloy and that there were not significant differences in the concentrations of all elements between Zone B, bulk and top. The solidification microstructures in the latter three areas were different, as shown in Figure 4. The Si and Al concentrations respectively increased from approx. 13 to 25.5 at.% and decreased from approx. 54 to 31 at.% from Zone A to Zone B. At the interface between Zone A and Zone B the Si concentration was about 36 at.%. At this interface the vol.% of Nb_5_Si_3_ was very high (Figure 4a). In the transition from Zone B to bulk the Si and Al concentrations respectively increased from approx. 25 to 27 at.% and decreased from approx. 31 to 29 at.%, i.e., the changes were minor and within the error of analysis. In other words, significant changes in the concentrations of Al and Si occurred in the bottom of the button and near the transition from Zone A to Zone B.

**Figure 6:**
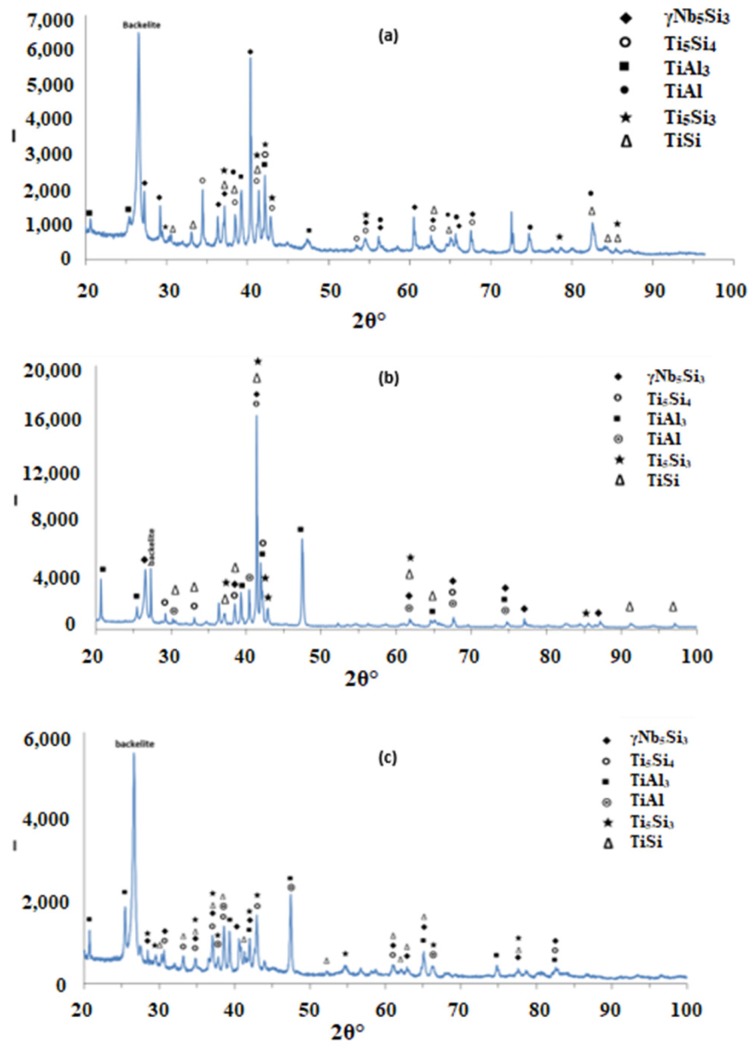
X ray diffractograms of the alloy Nb_1.3_Si_2.4_Ti_2.4_Al_3.5_Hf_0.4_ (**a**) as cast; (**b**) heat treated at 800 °C; (**c**) heat treated at 1200 °C.

According to the XRD data (Figure 6a) the aluminides TiAl and TiAl_3_ and the silicides Ti_5_Si_4_, Ti_5_Si_3_, TiSi and hexagonal γNb_5_Si_3_ were present in the microstructure. The quantitative analysis data confirmed the presence of all the above phases in all parts of the button with the exception of TiAl. The average composition of (Ti,Nb)Al_3_ (= 12.2Nb–2.4Si–12.7Ti–72.1Al–0.7Hf) did not differ significantly along the cross section, the Al + Si sum was about 75 at.% but the standard deviations of each element with the exception of Hf were greater than one in the zones A and B. The contrast exhibited by the tri-aluminide grains (Figure 4c) varied depending on their Al content. Also, the average composition of TiSi (= 12.2Nb–44.3Si–30.7Ti–6.6Al–6Hf) did not differ significantly along the cross section, the Al + Si sum was about 50 at.% but the standard deviations of each element with the exception of Hf were greater than one in the bulk and top. The TiSi formed a thin “layer” between Ti_5_Si_4_ and tri-aluminide (Figure 4d). At the very bottom of Zone A, meaning in the areas that had been in direct contact with the crucible wall, we did not observe the Ti_5_Si_4_ and TiSi around the Nb_5_Si_3_, but the latter two silicides were observed around Nb_5_Si_3_ further in Zone A (i.e., further away from the crucible wall). The average composition of Ti_5_Si_4_ was different in the bottom (12Nb–46.1Si–33.6Ti–2.3Al–6Hf) and in the top and bulk (18.2Nb–47Si–24.3Ti–1.7Al–8.7Hf). This silicide was noticeably poor in Al. The average composition of Nb_5_Si_3_ (= 22.3Nb–38.8Si–29.4Ti–4.6Al–4.9Hf) did not differ significantly along the cross section and had Nb/(Ti + Hf) ≈ 0.66 in the bottom, bulk and top but in the zones A and B we also observed Ti rich 5-3 silicide with Nb/(Ti + Hf) ≈ 0.36 with average composition 15Nb–38.2Si–38.8Ti–5.1Al–2.9Hf. In Figure 4 the silicides are indicated as 5-3.

Heat treated: After the 100 h heat treatments at 800 and 1200 °C the zones A and B were still observed in cross sections of the buttons. The actual compositions were 13.3Nb–23.2Si–23.6Ti–36.6Al–3.3Hf and 13.3Nb–21.6Si–23Ti–39.2Al–2.8Hf respectively for the 800 and 1200 °C heat treatment temperatures. These were the average compositions of all EDS analyses taken from the top, bulk and bottom of the heat treated buttons and were not significantly different from the actual composition of the cast alloy. The standard deviations for Si and Al were still high with that of the latter being higher, as was the case for the cast alloy. For the bulk and top of the heat treated button the standard deviations of all elements were smaller, and less and/or equal to one for Hf, Nb and Ti. The concentrations of each element in the bottom, bulk and top of the cast alloy are shown by the red and green colour vertical bars respectively for the 800 and 1200 °C temperatures in Figure 5.

According to the XRD data (Figure 6b,c) the aluminides TiAl and TiAl_3_ and the silicides Ti_5_Si_4_, Ti5Si_3_, TiSi and hexagonal γNb_5_Si_3_ were present in the microstructure. The presence of TiAl at both heat treatment temperatures was confirmed by quantitative EDS. The TiAl was observed in the areas in-between the 5-3 silicide grains where TMAl_3_ was also present. It was not easy to distinguish each phase using the contrast in back scatter electron imaging, because of the partitioning of Ti in both phases. The TiAl was scarcely present in the top and bulk of the button where at both temperatures the TiAl had a similar average composition (11.4Nb–3.2Si–24.7Ti–59Al–1.6Hf) with Al + Si between 62 and 66 at.%. However, no TiAl was observed in the bottom of the alloy that was heat treated at 800 °C and in the bottom of the button that was heat treated at 1200 °C the TiAl was very poor in Nb (2.3Nb–10.4Si–29.9Ti–55.1Al–2.3Hf). The TMAl_3_ had similar compositions for both temperatures with Al + Si between 73 and 75 at.% and with average composition similar to that given above for the as cast alloy. In the bottom, bulk and top of the alloy that was heat treated at 800 °C the Ti_5_Si_4_ silicide had a similar average composition (15.3Nb–45.6Si–30.6Ti–2Al–6.5Hf). However, after the heat treatment at 1200 °C the average compositions of the Ti_5_Si_4_ were different between the bottom (7.8Nb–46.5Si–38.5Ti–1.8Al–5.4Hf) and bulk and top (13.8Nb–45.2Si–32.4Ti–2.5Al–6.1Hf) and both were different from the as cast alloy but still poor in Al. With increasing heat treatment temperature, the Ti_5_Si_4_ became richer in Ti and poorer in Nb. At both heat treatment temperatures, the TiSi had similar composition with that given above for the as cast alloy. At 800 °C the Nb_5_Si_3_ had Nb/(Ti + Hf) ≈ 0.65 and average composition similar to the as cast alloy. However, at 1200 °C the 5-3 silicide had become richer in Ti with Nb/(Ti + Hf) ratios about 0.42 (17.3Nb–37.2Si–37.1Ti–4.1Al–4.1Hf) and 0.2 (9.7Nb–38.8Si–45.4Ti–2.8Al–3.2Hf).

**Figure 7:**
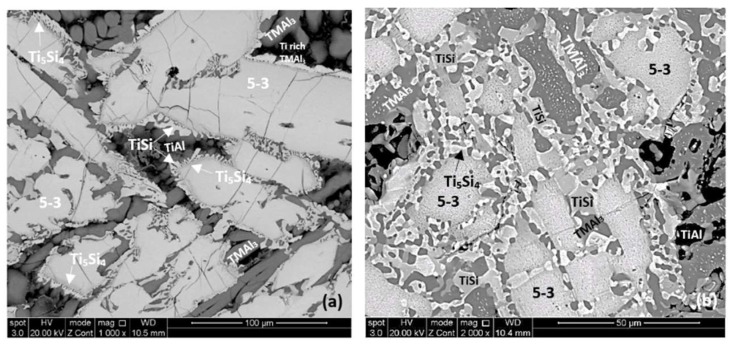
SEM backscatter electron images of the bulk microstructure of the heat treated alloy Nb_1.3_Si_2.4_Ti_2.4_Al_3.5_Hf_0.4_ (**a**) 800 °C, (**b**) 1200 °C.

The microstructures of the heat treated alloy are shown in Figure 7. After the heat treatment at 800 °C the microstructure had not changed significantly (compare Figure 4d and Figure 7a) but the 5-3 silicide grains exhibited severe cracking. After the heat treatment at 1200 °C the microstructure had changed considerably. There was precipitation of a second phase in the TMAl_3_ grains. This phase exhibited bright contrast under back scatter electron imaging and its identity is unknown. There was also precipitation of a second phase inside 5-3 grains. This phase was present as finer particles compared with those observed in the bulk of TMAl_3_ grains and their contrast was similar to that of TiSi. The latter had grown significantly compared with the cast microstructure. There was also growth of the Ti_5_Si_4_, some parts of which exhibited darker and others lighter contrast (owing to different Ti and Hf concentrations). Similar variations in contrast were also exhibited by 5-3 silicides depending on their Nb/(Ti + Hf) ratios.

### 4.3. Isothermal Oxidation 

#### 4.3.1. Alloy Nb_1.7_Si_2.4_Ti_2.4_Al_3_Hf_0.5_

**Figure 8:**
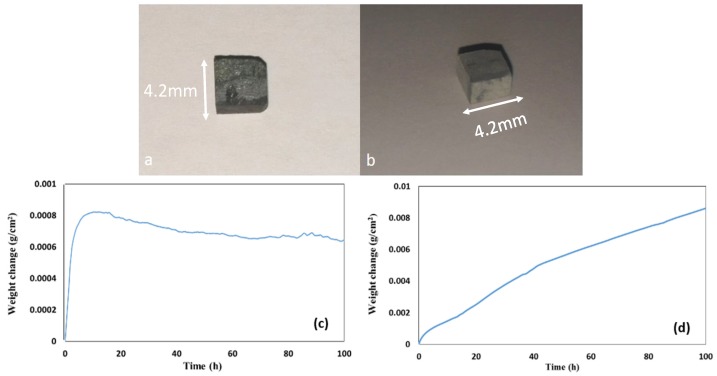
Oxidised specimens of the alloy Nb_1.7_Si_2.4_Ti_2.4_Al_3_Hf_0.5_ (**a**) at 800 °C and (**b**) at 1200 °C and weight change versus time data (**c**) at 800 °C and (**d**) at 1200 °C.

The oxidised specimens after 100 h isothermal oxidation at each temperature are shown in Figure 8a,b. At 800 °C the alloy formed a thin scale and lost weight 0.74 mg/cm^2^. At 1200 °C the alloy gained weight 8.5 mg/cm^2^ and formed a thicker scale (see Figure 8c,d).

**Figure 9:**
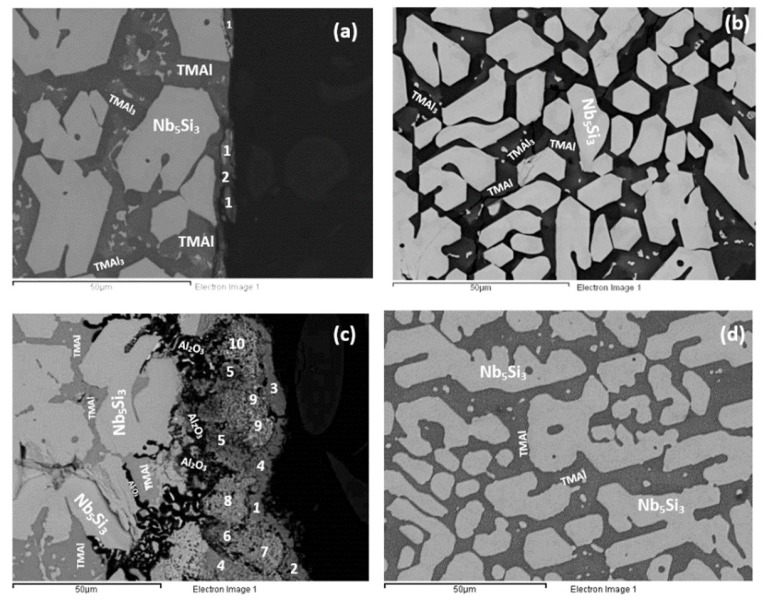
SEM backscatter electron images of the microstructure of the alloy Nb_1.7_Si_2.4_Ti_2.4_Al_3_Hf_0.5_ after isothermal oxidation (**a**,**b**) at 800 °C; (**c**,**d**) at 1200 °C. (**a**,**c**) scale and substrate below scale; (**b**,**d**) bulk. In (**a**) 1 is Si rich oxide with Al,Nb,Ti, and 2 is Al rich oxide with Nb,Si,Ti. In (**c**) 1, 2, 3 indicate Ti rich mixed oxides, 4, 5 indicate Al and Ti rich mixed oxides, 6, 7, 8, 9, 10 indicate Si and Nb rich mixed oxides.

**Figure 10:**
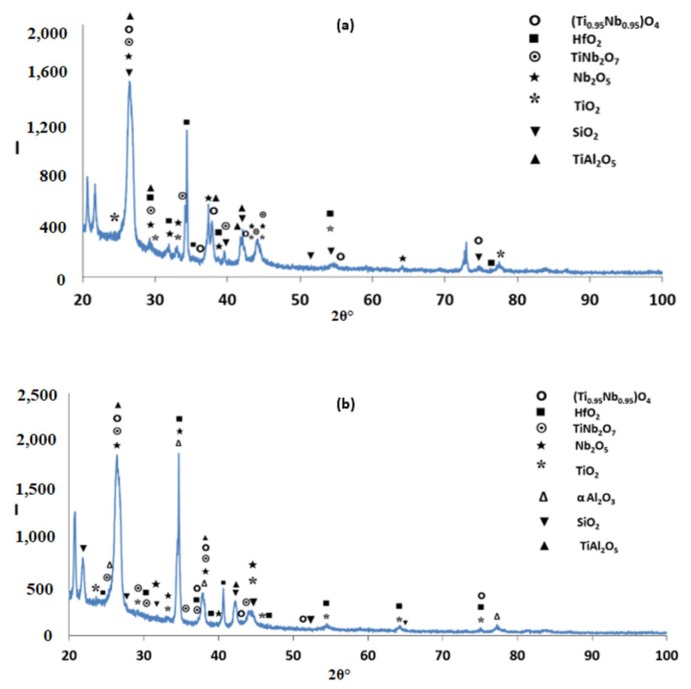
Glancing angle X ray diffractograms (θ = 5°) of the alloy Nb_1.7_Si_2.4_Ti_2.4_Al_3_Hf_0.5_ after isothermal oxidation (**a**) at 800 °C; (**b**) at 1200 °C.

**Figure 11:**
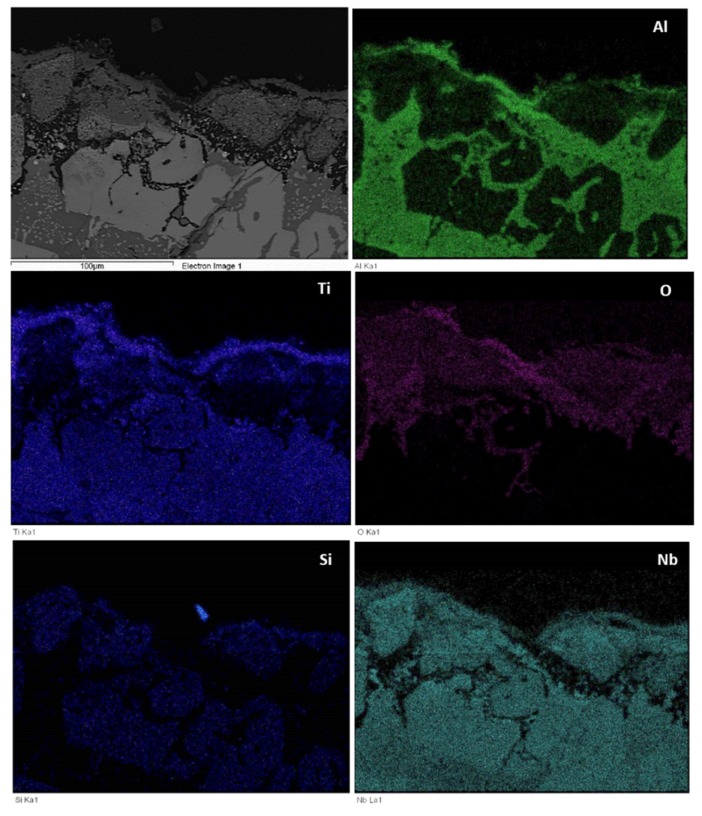

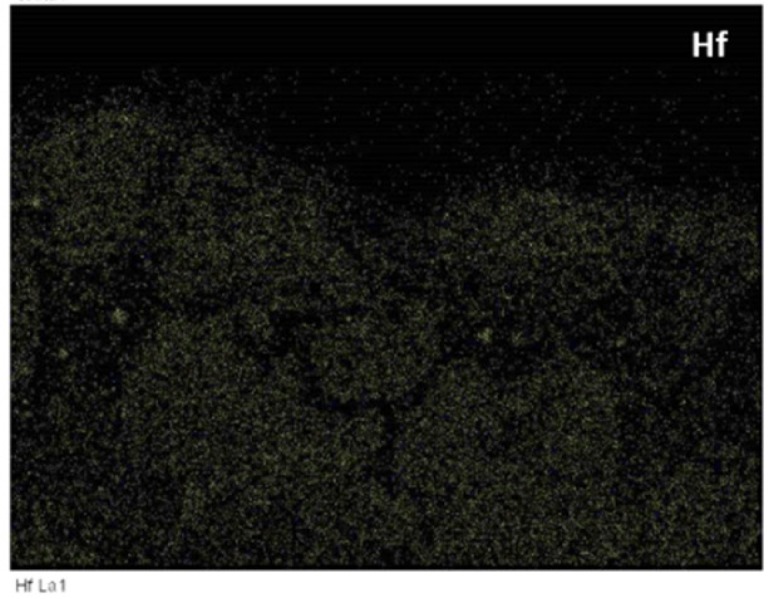
BSE image and X-ray elemental maps of scale formed on the alloy Nb_1.7_Si_2.4_Ti_2.4_Al_3_Hf_0.5_ at 1200 °C.

The microstructures just below the scale and in the bulk are shown in Figure 9. Figure 10 shows the glancing angle XRD data of the oxidised specimens. At 800 °C the XRD data suggested the presence of Ti niobates, Nb_2_O_5_, HfO_2_, TiO_2_, TiAl_2_O_5_ and SiO_2_. The back scatter electron (BSE) imaging and analysis data confirmed the presence of discontinuous thin scale consisting of Al containing mixed oxides, see Figure 9a. At 1200 °C the XRD data suggested the presence of the same oxides plus αAl_2_O_3_. Figure 9c and Figure 11 show a thicker “layered” scale that consisted of Ti-rich mixed oxide and Al and Ti rich mixed oxide at the top, beneath formed a Nb and Si-rich mixed oxide, beneath was Al and Ti rich mixed oxide and beneath the latter was a continuous mixture of Al_2_O_3_ (major phase) with dispersed oxide(s). The identity of the latter is not known. The dispersed oxide(s) exhibited a contrast similar to that of the Ti-rich oxides. There was also internal oxidation with Al_2_O_3_ forming at interfaces between Nb_5_Si_3_ and (Ti,Nb)Al (see Figure 9c and Figure 11).

At both temperatures the Nb_5_Si_3_, TiAl and TMAl_3_ were contaminated by oxygen both below the scale and in the bulk of the oxidised specimens. The contamination of Nb_5_Si_3_ was more severe than that of the TiAl. The TMAl_3_ exhibited the lower contamination. At 1200 °C the contamination by oxygen of Nb_5_Si_3_ increased and the contamination by oxygen of the aluminides had not changed significantly.

#### 4.3.2. Alloy Nb_1.3_Si_2.4_Ti_2.4_Al_3.5_Hf_0.4_

**Figure 12:**
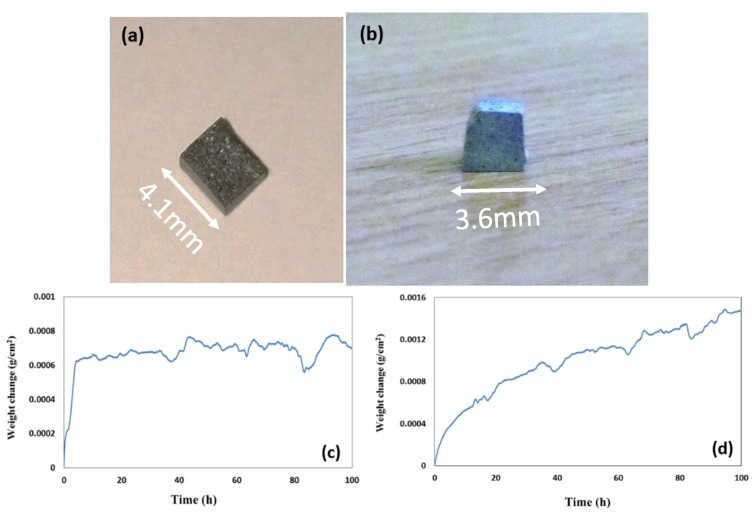
Oxidised specimens of the alloy Nb_1.3_Si_2.4_Ti_2.4_Al_3.5_Hf_0.4_ (**a**) at 800 °C and (**b**) at 1200 °C and weight change versus time (**c**) at 800 °C and (**d**) at 1200 °C.

**Figure 13:**
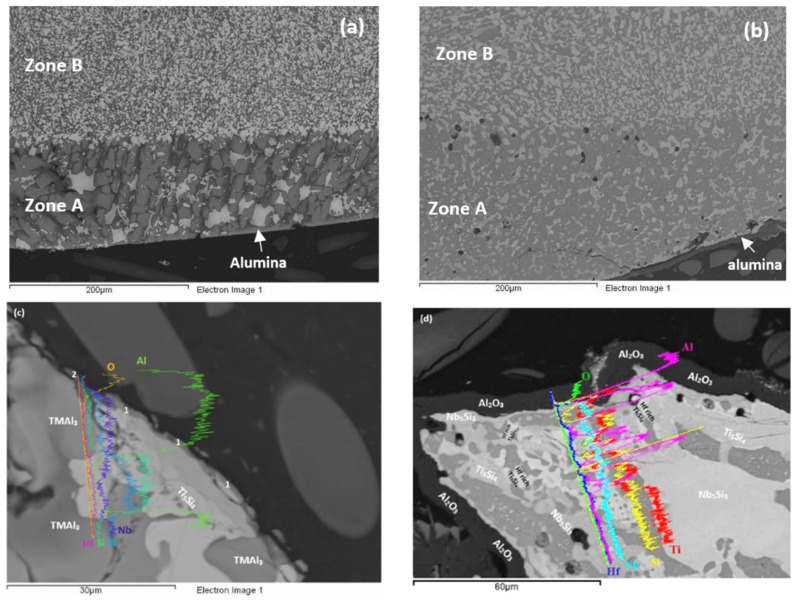
SEM back scatter electron images of cross sections of oxidised specimens of the alloy Nb_1.3_Si_2.4_Ti_2.4_Al_3.5_Hf_0.4_ (**a**) at 800 °C and (**b**) at 1200 °C; (**c**) line scan at 800 °C, 1 indicates Si containing Nb and Ti mixed oxide, 2 indicates Al_2_O_3_; (**d**) line scan at 1200 °C.

The oxidised specimens of this alloy after 100 h isothermal oxidation at each temperature are shown in Figure 12a,b. At 800 °C the alloy formed a thin scale (Figure 13a,c) and gained weight 0.68 mg/cm^2^. At 1200 °C it gained weight 2.6 mg/cm^2^, followed parabolic oxidation kinetics with the rate constant *K*_p_ = 1 × 10^−11^ g^2^ cm^−4^ s^−1^, an order of magnitude lower than that of the alloy Nb_1.7_Si_2.4_Ti_2.4_Al_3_Hf_0.5_ and formed a continuous alumina scale (see Figure 13b,d).

**Figure 14:**
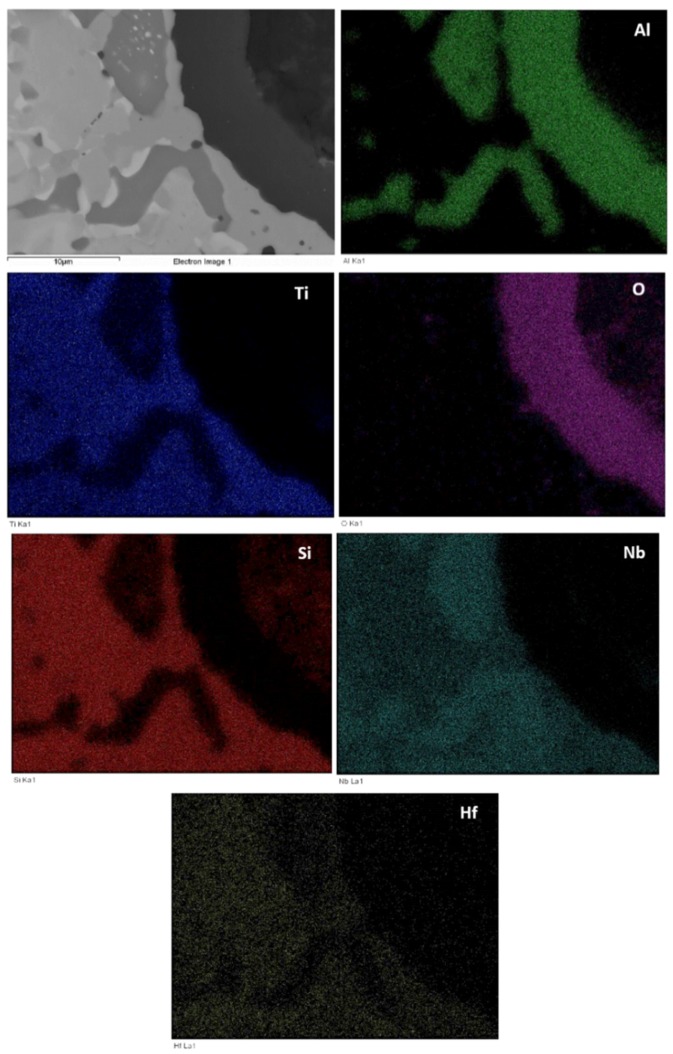
BSE image and X-ray elemental maps of scale formed on the alloy Nb_1.3_Si_2.4_Ti_2.4_Al_3.5_Hf_0.4_ at 1200 °C.

Cross sections of oxidised specimens are shown in Figure 13. At 800 °C a thin scale formed that consisted of “islands” of alumina, and Si containing mixed oxides (Figure 13a,c). At 1200 °C a thicker continuous alumina scale was formed, see Figure 13b,d. Figure 13c shows alumina formed on tri-aluminide grain and Figure 13a shows continuous thin alumina that formed on an area rich in tri-aluminide. Figure 13d shows thick continuous Al_2_O_3_ scale grown on a larger specimen that was oxidised in a muffle furnace at 1200 °C. Figure 14 shows that at 1200 °C the alumina scale was formed on top of γNb_5_Si_3_, while in other parts (not shown) also it was formed on top of Si rich or Al rich intermetallics. In some parts there was a very thin Ti rich oxide on top of the 5 μm thick alumina (see Figure 13d). The microstructures in the bulk of these specimens were similar to those shown in Figure 7. In the bulk the contamination of the Nb_5_Si_3_ was slightly lower and of TiAl and TMAl_3_ was similar to that of the same compounds in the alloy Nb_1.7_Si_2.4_Ti_2.4_Al_3_Hf_0.5_.

**Figure 15:**
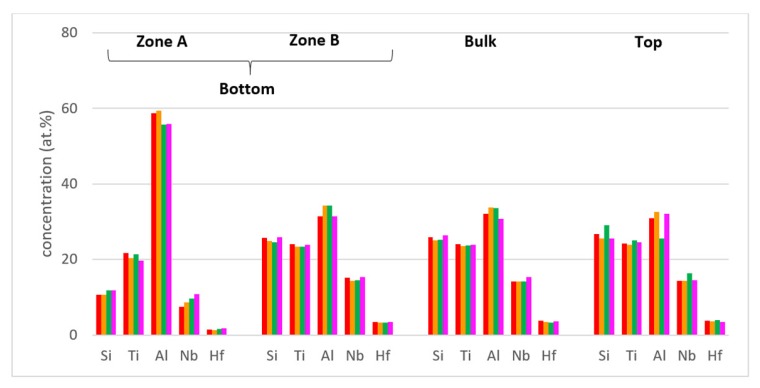
Comparison of the concentrations of elements in the bottom, bulk and top of the alloy Nb_1.3_Si_2.4_Ti_2.4_Al_3.5_Hf_0.4_. Heat treated at 800 °C—red bars (the same as in Figure 5), heat treated at 1200 °C—green bars (the same as in Figure 5), oxidised at 800 °C—orange bars, oxidised at 1200 °C—pink bars.

Figure 15 shows the compositions of the different areas after the heat treatments at 800 and 1200 °C (red and green bars, these are the same as in Figure 5) and after the isothermal oxidation at the same temperatures. It shows small changes in Al concentrations in Zone B, the bulk and top and small decrease and increase, respectively, of Ti and Nb, in Zone A.

The average compositions of the phases at 800 °C were the same as after the heat treatment at the same temperature with the exception of the Ti_5_Si_4_ compound that became poorer (11.6Nb–46.8Si–32.1Ti–1.4Al–8Hf) and richer (17.1Nb–45.5Si–28.6Ti–2.5Al–6.2Hf) in Nb respectively in the bulk and top and in the bottom of the oxidised alloy. The average compositions of the phases at 1200 °C were the same with those after the heat treatment at the same temperature with the exception of (Ti,Nb)Al_3_ which became richer and poorer respectively in Nb and Ti in both the bottom (12.7Nb–1.1Si–13.8Ti–71.6Al–0.7Hf) and bulk and top (17.7Nb–2.6Si–9.8Ti–69.3Al–0.6Hf) and the Ti_5_Si_4_ which became richer and poorer respectively in Nb and Ti in the bottom and bulk and top with essentially the same composition throughout the oxidised alloy (15.9Nb–46.3Si–28.6Ti–1.8Al–7.4Hf). The Ti_5_Si_4_ silicide in the microstructures of the oxidised specimens at 800 and 1200 °C continued to be poor in Al.

**Figure 16:**
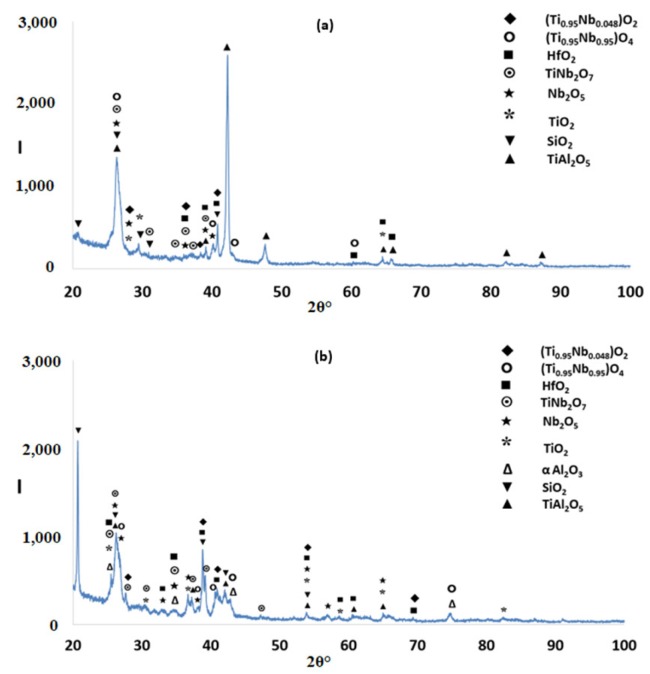
Glancing angle X ray diffractograms (θ = 5°) of the alloy Nb_1.3_Si_2.4_Ti_2.4_Al_3.5_Hf_0.4_ after isothermal oxidation (**a**) at 800 °C; (**b**) at 1200 °C.

The glancing angle XRD data in Figure 16 shows (i) that at 800 °C the scale consisted of Ti niobates and TiO_2_, SiO_2_, TiAl_2_O_5_, Nb_2_O_5_, HfO_2_ (Figure 16a) and (ii) that the same oxides were present at 1200 °C plus αAl_2_O_3_ (Figure 16b). Compared with the Nb_1.7_Si_2.4_Ti_2.4_Al_3_Hf_0.5_ alloy (i) the glancing angle XRD had peaks that corresponded only to the aluminium titanate (TiAl_2_O_5_) and (ii) the weight gain versus time data for both temperatures showed that the scale was not stable. Indeed, there were sudden changes in weight gain, particularly at 800 °C, compare Figure 8c,d with Figure 12c,d.

## 5. Discussion

### 5.1. Macrosegregation

The as cast microstructures of both alloys were chemically inhomogeneous. Table 1 compares the parameters that describe macrosegregation of Si (MACSi) in the two alloys with those of the alloy NbSiTiHf-5Al (nominal composition Nb–24Ti–18Si–5Hf–5Al, [20]). In Reference [20] it was shown that Al increased MACSi and MACTi (macrosegregation of Ti) and that the chemical inhomogeneity of these elements persisted after heat treatment, which is supported by the results of this work. The data in Table 1 shows that MACSi increased as the parameters *T*_m_, Δ*H*_m_, Δ*H*_m_^sd^, *T*_m_^sd^ decreased and the parameters Δ*H*_m_/*T*_m_, Δ*H*_m_^sp^, *T*_m_^sp^ increased, in agreement with [27]. Note that the alloy Nb_1.3_Si_2.4_Ti_2.4_Al_3.5_Hf_0.4_ has the lowest T_m_ of the three alloys, which increases the likelihood of forming a deeply undercooled melt in areas of high cooling rate (see Section 2 and [27]). Moreover, it has the highest Δ*H*_m_/*T*_m_ value, which may indicate an increased difficulty for the growth of intermetallic compounds in the alloy Nb_1.3_Si_2.4_Ti_2.4_Al_3.5_Hf_0.4_ (see Section 5.2.2).

### 5.2. Microstructures

#### 5.2.1. Alloy Nb_1.7_Si_2.4_Ti_2.4_Al_3_Hf_0.5_

The solid solution was not stable, as required by the alloy design criteria (see Section 2). The cast microstructure in the bottom of Nb_1.7_Si_2.4_Ti_2.4_Al_3_Hf_0.5_ consisted of hexagonal Ti rich Nb_5_Si_3_ and tri-aluminide (Ti,Nb)Al_3_ (see Section 4.1). As the Ti rich 5-3 silicide formed the melt became lean in Si, Ti, Hf and Nb, and rich in Al. As the solidification proceeded, from the aforementioned melt formed the tri-aluminides with different transition metal content depending on the local melt chemistry. As the tri-aluminides formed, the melt became lean in Al and rich in Si and Nb, and the concentration of Ti in the melt either increased or did not change depending on the chemistry of the tri-aluminide. In the latter melt formed Ti and/or Hf rich Nb_5_Si_3_ and the melt near the silicide became lean in Si, Hf and Nb, rich in Al and either lean or rich in Ti depending on the chemistry of Nb_5_Si_3_. As the TiAl formed in this melt the latter became lean in Al, rich in Si, Hf and Nb with no significant change in Ti concentration. In this Si rich melt formed the Ti_2.35_Al_1.65_Si compound and then Ti_2_Al_5_ and thus the melt became rich in Ti, Nb, Hf and Si and lean in Al. As the solidification proceeded towards the top of the button, from the latter melt formed Nb_5_Si_3_ and the surrounding melt became lean in Si, Hf and Nb and rich in Al and Ti. Then the TiAl formed and the melt became lean in Al, rich in Si, Hf and Nb with no significant change in Ti concentration, and in this Si rich melt formed the TM_3.7_Al_3_Si compound. The solidification sequence discussed above indicates hexagonal Nb_5_Si_3_ as the primary phase. If the alloy Nb_1.7_Si_2.4_Ti_2.4_Al_3_Hf_0.5_ is considered as a (Ti,Nb,Hf)–Al–Si alloy, the above conclusion is in agreement with the liquidus projection of the Ti–Al–Si system [25] which shows that the average alloy composition is in the Ti_5_Si_3_ phase area. The formation of the tri-aluminide from the melt surrounding the hexagonal Nb_5_Si_3_ is in agreement with the Ti–Al–Si solidus projection [25]. Thus, based on the experimental results and the above discussion it is suggested that the solidification path in the bottom of the alloy was L → L + γNb_5_Si_3_ → L + γNb_5_Si_3_ + TMAl_3_, in the bulk L + γNb_5_Si_3_ + βNb_5_Si_3_ → L + γNb_5_Si_3_ + βNb_5_Si_3_ + TiAl + Ti_2.35_Al_1.65_Si + Ti_2_Al_5_ and in the top L + βNb_5_Si_3_ → L + βNb_5_Si_3_ + TiAl + TM_3.7_Al_3_Si + (βNb_5_Si_3_ + TiAl)_eutectic_.

The phases present in the microstructure of the heat treated alloy that were confirmed by both the XRD and EDS data were tetragonal βNb_5_Si_3_, hexagonal γNb_5_Si_3_, TMAl_3_, TiAl and possibly Ti_2_Al_5_. This is in agreement with the 1250 °C isothermal section of the Ti–Al–Si system [25]. The composition of the Nb_5_Si_3_ had changed towards lower Nb/(Ti+Hf) ratios, which would suggest that the hexagonal Nb_5_Si_3_ is likely to be the stable 5-3 silicide in this alloy. The same was concluded for the Nb_5_Si_3_ in the alloy NbSiTiHf-5Al in Reference [20]. In the aluminides, the Si concentration was reduced to very low levels, which were in agreement with the literature.

#### 5.2.2. Alloy Nb_1.3_Si_2.4_Ti_2.4_Al_3.5_Hf_0.4_

The solid solution also was not stable in this alloy, as required by the alloy design criteria (see Section 2). The same phases were present in all parts of the button of the alloy, namely the hexagonal γ(Nb,Ti)_5_Si_3_, the (Ti,Nb)_5_Si_4_, (Ti,Nb)Si silicides and (Ti,Nb)Al_3_ aluminide. The vol.% of TMAl_3_ was significantly higher in Zone A compared with Zone B, bulk and top of the button.

According to the Ti-Si binary phase diagram, in Si rich melts where, as the solidification starts, the Ti_5_Si_3_ is the primary phase there is a “cascade” of peritectic reactions, namely L + Ti_5_Si_3_ → Ti_5_Si_4_, then L + Ti_5_Si_4_ → TiSi. The microstructure of such a Si rich alloy would consist of the Ti_5_Si_3_ (primary) “surrounded” by the Ti_5_Si_4_ (first peritectic) and then Ti_5_Si_4_ “surrounded” by TiSi (second peritectic). This was observed in the alloy Nb_1.3_Si_2.4_Ti_2.4_Al_3.5_Hf_0.4_ (Figure 4d).

The tri-aluminide formed in the areas between the “composite” silicide grains (composite here means Nb_5_Si_3_ (5-3) core surrounded by Ti_5_Si_4_, surrounded by TMSi), i.e., in the last melt to solidify. Thus, it was deduced that in the alloy Nb_1.3_Si_2.4_Ti_2.4_Al_3.5_Hf_0.4_ the melting temperatures of the alloyed Nb_5_Si_3_, Ti_5_Si_4_ and TMSi silicides were higher than the tri-aluminide TMAl_3_.

As the primary Nb_5_Si_3_ formed the surrounding melt became poor in Hf, Nb, Si and Ti and rich in Al, from this melt the Ti_5_Si_4_ formed around the 5-3 silicide via a peritectic reaction and the melt became poor in Hf, Si, Ti and richer in Al and Nb. Then from this melt the TiSi formed around the Ti_5_Si_4_ via a peritectic reaction and from Al rich and Si and Hf poor melt formed the TMAl_3_. It is suggested that the solidification path of the alloy in Zone B, bulk and top of the button was L → L + γNb_5_Si_3_ then L + γNb_5_Si_3_ → TM_5_Si_4_, then L + TM_5_Si_4_ → TMSi → γNb_5_Si_3_ + TM_5_Si_4_ + TMSi + TMAl_3_.

The average composition of Zone A was different than those of Zone B and the bulk and top (Figure 5). In the deeply undercooled melt next to the crucible wall the peritectic reactions that would result in the growth of Ti_5_Si_4_ and TiSi around the primary phase were suppressed. The melt surrounding the primary phase became less poor in Si and richer in Al than it would have been had the peritectic reactions occurred. Thus, as the primary Nb_5_Si_3_ nucleated and grew in the undercooled melt, the melt became rich in Al and poor in Nb, Si and Ti and from this melt formed the TMAl_3_ making the melt poor in Al and rich in Si. The vol.% of TMAl_3_ was very high in Zone A owing to the chemical composition of the latter, thus the melt became very rich in Si and poor in Al as the solidification advanced away from the cold crucible wall.

It is reasonable to assume that the growth velocity *V*_S/L_ was “constant” during the solidification of Zone A (growth velocity “imposed” by the conditions near the crucible wall). The model of Tiller et al. [39] for the solute concentration (*C*_L_*) at the S/L interface during the initial transient solidification shows that the *C*_L_* is proportional to the solute concentration in the melt *C*_o_. The undercooling Δ*T*_CS_ during the initial transient is given by the equation [40],

Δ*T*_CS_ = *m*_L_ [*C*_o_/*k*_o_](1 − *k*_o_)[1 − exp(−*k*_o_*V*_S/L_^2^*t*/*D*_L_)][1 − exp(−*V*_S/L_*x*/*D*_L_)] – *Gx*

where *t* is time, *x* is the distance from S/L interface, *D*_L_ is diffusion coefficient in the melt, *k*_o_ is the partition coefficient, *G* is the temperature gradient and *m*_L_ is the liquidus slope. In other words, as the melt became rich in Si ahead of the advancing S/L front (i.e., *C*_o_ increased in the above equation) the Δ*T*_CS_ increased. Thus, as solidification proceeded in Zone A and latent heat was released from the solidifying compounds, the undercooling of the melt was enough to ensure the growth of the intermetallic compounds that formed in this zone, and as the thickness of the latter increased the melt ahead of the advancing solidification front became richer and richer in Si until it reached the concentration Si ≈ 36 at.% and a high vol.% of Nb_5_Si_3_ formed. The latter resulted in the transition from Zone A to Zone B (see Section 4.2 and Figure 4a and Figure 13a).

If the alloy is considered as an alloy of the (Ti,Nb,Hf)–Si–Al system, primary hexagonal 5-3 silicide is in agreement with the results reported in Reference [35]. If the alloy is considered as an alloy of the (Nb,Hf)-Ti-(Si,Al) system, then according to Bulanova and Fartushna [41] the reaction L + (Ti,Nb)_5_Si_3_ → β(Nb,Ti)_5_Si_3_ + (Ti,Nb)_5_Si_4_ occurs at *T* < 1815 °C and then via L + β(Nb,Ti)_5_Si_3_ → (Ti,Nb)_5_Si_4_ + (Nb,Ti)Si_2_ and L + (Ti,Nb)_5_Si_4_ → (Nb,Ti)Si_2_ + (Ti,Nb)Si the TMSi is formed below 1570 °C. Note that we did not observe (Nb,Ti)Si_2_ in the alloy Nb_1.3_Si_2.4_Ti_2.4_Al_3.5_Hf_0.4_. The above would suggest that in the latter the formation of the TMAl_3_ tri-aluminide was “controlled” by the Ti–Al–Si phase equilibria and that of the TMSi silicide by the Nb–Ti–Si phase equilibria.

A characteristic feature of the microstructures observed in the cast, heat treated and oxidised conditions was the fibrous nature (structure) of Ti_5_Si_4_, see Figure 4d and Figure 7. Similar structure for Ti_5_Si_4_ has been reported by Gupta [42] and Park et al. [37]. Gupta described it as “wool like” and observed it in Ti–Al–Si diffusion couples air cooled after annealing at 800 or 900 °C for 3 or 6 h. The couples were made from pure Ti and eutectic Al–Si alloy. In the couples studied by Gupta the fibrous Ti_5_Si_4_ “grew” towards (“was over”, “formed in a matrix of”) TiAl_3_ and was on top of (“sitting on”) Ti_5_Si_3_ that exhibited a brighter contrast than Ti_5_Si_4_. The average Al concentration in Ti_5_Si_4_ given by Gupta [42] was approximately 7.5 to 8 at.%, and is higher than the average Al concentrations (about 2 at.%) analysed in this research, see Section 4.2, and the concentration reported in Reference [17] (about 0.2 to 0.4 at.% Al). Gupta did not observe TiSi in his diffusion couples.

Park et al. [37] also reported about the formation of Ti_5_Si_4_ in diffusion couples annealed at 1100 °C for ≥ 200 h. They studied two couple types, one between TiAl and TiSi_2_ (type A, our notification) and the other was TiAl/Ti/TiSi_2_ (type B, our notification). The type A and type B couples were referred to respectively as “direct interface reaction” and “biased interface reaction” couples by Park et al. [37]. In the type A couple the sequence of phases was TiAl/TiAl_2_/Ti_2_Al_5_/TiAl_3_ + Ti_5_Si_4_/Ti_5_Si_4_/TiSi/TiSi_2_, i.e., the Ti_5_Si_3_ silicide did not form. The Ti_5_Si_4_ formed its own irregular “thick porous” layer. The solubility of Al was < 2 at.%. In our work the average Al concentrations in the Ti_5_Si_4_ in the cast and heat treated (800 and 1200 °C) alloy were ≈ 2 at.%. The Ti_5_Si_4_ also grew a columnar morphology through TiAl_3_ and for this growth morphology, according to Park et al. [37], the rate-limiting component was Ti (*D*_Ti(TiAl3)_ < *D*_Si(Ti5Si4)_, where D*_i_* is diffusivity of species *i* (= Si,Ti) in the indicated intermetallic).

In the type B couple, the Ti_5_Si_3_ silicide formed. The Ti_5_Si_4_ also formed but it was not porous. The sequence of phases was TiAl/Ti_3_Al/Ti/Ti_3_Si/Ti_5_Si_3_/Ti_5_Si_4_/TiSi/TiSi_2_. In the Ti_5_Si_3_ the Al concentration was about 5 at.%, the same as the average Al concentrations measured in this work in hexagonal 5-3 silicide in the cast, heat treated and oxidised alloy, see Section 4.2. The Al concentration in Ti_5_Si_4_ was the same as in the type A couple. Park et al. [37] suggested that in the type A couple the Ti flux was not enough for the formation of Ti_5_Si_3_.

In this work, in the alloy Nb_1.3_Si_2.4_Ti_2.4_Al_3.5_Hf_0.4_ the Ti_5_Si_4_ was observed in the cast, heat treated and oxidised conditions to be in contact with Ti_5_Si_3_, with TMSi, and with TMAl_3_. The Ti_5_Si_4_ formed a fibrous (columnar, wool like) structure. The formation of Ti_5_Si_4_ and the other phases was accompanied by partitioning of solute during solidification and solid state cooling of the ingot, and also during each heat treatment and during each oxidation experiment. Porosity was not observed in any of the 5-3/5-4/TMSi microstructures in this work. Thus, on the basis of the results of this work and those of Gupta [42] and Park et al. [37], it is concluded that in the alloy Nb_1.3_Si_2.4_Ti_2.4_Al_3.5_Hf_0.4_ the Ti_5_Si_4_ grew with a fibrous (columnar, wool like) morphology towards TMAl_3_ and that its growth was not accompanied by the formation of porosity.

The microstructure of the alloy after the heat treatment at 800 °C consisted of the hexagonal γNb_5_Si_3_, the aluminides TiAl and TMAl_3_ and the silicides Ti_5_Si_4_ and TMSi. Compared with the cast alloy, the TiAl was the new phase to form. The solubilities of Si and Al respectively in TiAl and Ti_5_Si_4_ were in agreement with the literature [35,37] but the solubility of Al in TMSi was higher than that reported by Park et al. [37]. The solubilities of Al, Hf and Ti in the Nb_5_Si_3_ were in agreement with the literature.

The microstructure of the alloy after the heat treatment at 1200 °C consisted of the hexagonal γNb_5_Si_3_, the aluminides TiAl and TMAl_3_ and the silicides Ti_5_Si_4_ and TMSi. Compared with the cast alloy, the TiAl was the new phase to form. Considering the results for the heat treatment at 800 °C it was concluded that the TiAl is a stable phase in this alloy. This conclusion is supported by the 1200 °C isothermal section for Ti-Al-Si in Reference [25], which shows that the average alloy composition falls in the three phase Ti_5_Si_3_, TiAl and TiAl_3_ area. The Al and Si concentrations respectively in Ti_5_Si_4_ and TiSi, and TiAl were close to those reported in the literature.

In the Nb_5_Si_3_ cracks were observed growing from one side of a grain to the other and often these cracks were parallel to each other, see Figure 7. In the cracked 5-3 silicide grains “lines” (sometimes curved) of darker contrast were observed mainly after the heat treatment at 800 °C (Figure 7) and a few were also observed after the heat treatment at 1200 °C. In the heat treated microstructure at 800 °C the growth of Ti_5_Si_4_ towards TMAl_3_ was noticeable (see above discussion, also Park et al. [37] reported that the growth rate of Ti_5_Si_4_ is higher than that of TiSi, TiAl_3_ and TiAl_2_ (decreasing growth rate sequence) with Ti_2_Al_5_ having the lowest growth rate from the aforementioned intermetallics). Furthermore, inside the Nb_5_Si_3_ and TMAl_3_ grains in the microstructure that was heat treated at 1200 °C there was evidence of precipitation of second phase(s), Figure 7b. Such precipitation was not observed at 800 °C.

The cracking of Nb_5_Si_3_ was attributed (a) to the enhanced anisotropy of the coefficient of thermal expansion with partitioning of Ti in the Nb_5_Si_3_ [43] and (b) to the large volume changes at the interfaces where the Ti_5_Si_4_ was formed [37]. The dark contrast in between the fibrous Ti_5_Si_4_ was the same as that exhibited by the tri-aluminide, but owing to the size of the growth features the latter could not be confirmed.

Agreement and/or disagreement with the literature regarding the Al concentration in Ti_5_Si_4_ and Nb_5_Si_3_ silicides was discussed above. The solubility of Si in TMAl_3_ (less than 3 at.% in the cast alloys Nb_1.7_Si_2.4_Ti_2.4_Al_3_Hf_0.5_ and Nb_1.3_Si_2.4_Ti_2.4_Al_3.5_Hf_0.4_ and even lower in the heat treated alloys, respectively less than 1 and 3 at.%) is in agreement with Bulanova et al. [35], lower than the range reported by Park et al. [37] (up to 7 at.%) and significantly lower than the values reported by Gupta [42] (9.2 to 14.3 at.%). There are no reports about the solubility of Al in TiSi in higher order systems than the ternary Ti-Al-Si where it is suggested to be very low or negligible [35,37]. However, the 700 and 1200 °C isothermal sections for the Ti-Al-Si system by Perrot [36] show solubility of Al in TiSi (about 10–12 at.% at 700 °C and about 5–7 at.% at 1200 °C). In this work, the solubility of Al in TiSi was about 16.7 at.% at 800 °C and 12.2 at.% at 1200 °C.

#### 5.2.3. Comparison with High Entropy Alloys and Nb–Silicide Based Alloys

The actual compositions of the alloys Nb_1.7_Si_2.4_Ti_2.4_Al_3_Hf_0.5_ and Nb_1.3_Si_2.4_Ti_2.4_Al_3.5_Hf_0.4_ met the “standard definition” of HEAs. Furthermore, the average compositions of the bottom, bulk and top of the alloy Nb_1.7_Si_2.4_Ti_2.4_Al_3_Hf_0.5_ and of Zone B, bulk, and top of the alloy Nb_1.3_Si_2.4_Ti_2.4_Al_3.5_Hf_0.4_ met the “standard definition” of HEAs but not Zone A of the latter alloy.

The parameters Δχ, δ and VEC of the alloys Nb_1.7_Si_2.4_Ti_2.4_Al_3_Hf_0.5_ and Nb_1.3_Si_2.4_Ti_2.4_Al_3.5_Hf_0.4_ respectively were in the following ranges: 0.1464 < Δχ < 0.158, 8.6552< δ < 9.5275, 3.808 < VEC < 3.97 and 0.1063 < Δχ < 0.1632, 6.4897 < δ < 10.1143, 3.483 < VEC < 3.909. The VEC of both alloys was outside the range of VEC values for bcc solid solution plus intermetallic(s) HEAs and outside the range of VEC values for Nb–silicide based alloys [44]. Both alloys had their δ values within the ranges of bcc solid solution plus intermetallic(s) HEAs. The parameter δ of the alloy Nb_1.7_Si_2.4_Ti_2.4_Al_3_Hf_0.5_ was within the range of values for Nb–silicide based alloys but for the alloy Nb_1.3_Si_2.4_Ti_2.4_Al_3.5_Hf_0.4_ the δ values of Zone A were outside the lower end of the range for Nb–silicide based alloys [44].

The parameter Δχ of the alloy Nb_1.7_Si_2.4_Ti_2.4_Al_3_Hf_0.5_ was within the range for bcc solid solution plus intermetallic(s) HEAs and within the range for Nb–silicide based alloys [44] and also was within the “forbidden range” of Δχ values for the Nb_ss_ in the latter alloys [1,44]. The parameter Δχ of the alloy Nb_1.3_Si_2.4_Ti_2.4_Al_3.5_Hf_0.4_ was outside the lower range of values for bcc solid solution and intermetallic(s) HEAs and Nb–silicide based alloys [1,44]. Furthermore, Zone A of the alloy Nb_1.3_Si_2.4_Ti_2.4_Al_3.5_Hf_0.4_ had Δχ values outside the range for bcc solid solution plus intermetallic(s) HEAs and Nb–silicide based alloys [44] and the Δχ values of this alloy were within the “forbidden range” of Δχ values for the Nb_ss_ in Nb–silicide based alloys [1,44] with the exception of Zone A.

#### 5.2.4. “Layered” Structure

**Figure 17:**
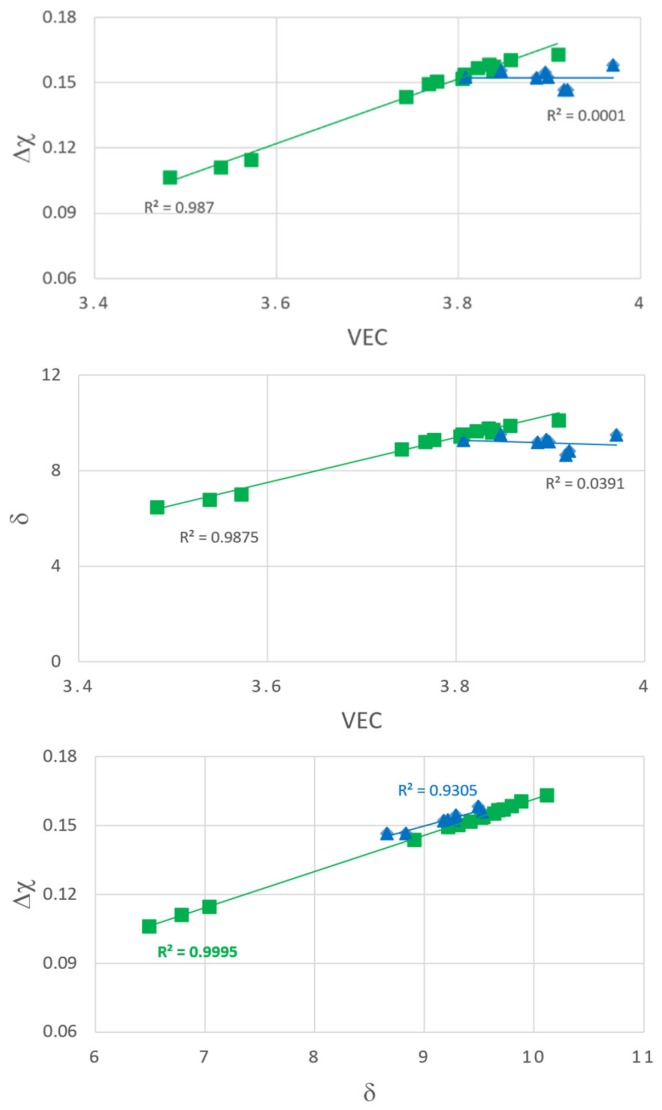
Plots of the parameters valence band (VEC), δ and Δχ for the microstructures of the cast and heat treated alloys Nb_1.7_Si_2.4_Ti_2.4_Al_3_Hf_0.5_ (blue triangle) and Nb_1.3_Si_2.4_Ti_2.4_Al_3.5_Hf_0.4_ (green squares).

Let us now return to the microstructures exhibited by the cross sections of the buttons of the alloys Nb_1.7_Si_2.4_Ti_2.4_Al_3_Hf_0.5_ and Nb_1.3_Si_2.4_Ti_2.4_Al_3.5_Hf_0.4_. Figure 17 shows plots of the parameters VEC, δ and Δχ for the microstructures of the cast and heat treated Nb_1.7_Si_2.4_Ti_2.4_Al_3_Hf_0.5_ (blue triangles) and Nb_1.3_Si_2.4_Ti_2.4_Al_3.5_Hf_0.4_ (green squares) alloys. Both alloys had similar concentrations of Hf, Si and Ti and both exhibited macrosegregation of Si that was more severe in the alloy Nb_1.3_Si_2.4_Ti_2.4_Al_3.5_Hf_0.4_ (see Table 1). The microstructure of the latter alloy also was sensitive to solidification conditions and was “layered” from bottom to top. This microstructure exhibited strong correlations (*R*^2^ > 0.987) between the parameters Δχ and VEC, δ and VEC and Δχ and δ (Figure 17) and also “sampled” a wider range of values of each parameter compared with the alloy Nb_1.7_Si_2.4_Ti_2.4_Al_3_Hf_0.5_. The microstructure of the latter was not layered from bottom to top and exhibited no correlations (*R*^2^ < 0.039) between the parameters δ and VEC and Δχ and VEC but there was a strong correlation between its parameters Δχ and δ with the data essentially parallel to that of the alloy Nb_1.3_Si_2.4_Ti_2.4_Al_3.5_Hf_0.4_ (Figure 17). Thus, the key to the “layering” of the microstructure of the alloy Nb_1.3_Si_2.4_Ti_2.4_Al_3.5_Hf_0.4_ was (i) its strong chemical inhomogeneity leading to severe macrosegregation of Si; (ii) its solidification that allowed it to sample (“experience”, “be exposed to”) a wide range of values of the parameters VEC, Δχ and δ that were strongly related to each other and (iii) its unique δ versus VEC and Δχ versus VEC relationships, which the alloy Nb_1.7_Si_2.4_Ti_2.4_Al_3_Hf_0.5_ could not form (Figure 17). It is concluded that the critical parameter of the alloy Nb_1.3_Si_2.4_Ti_2.4_Al_3.5_Hf_0.4_ was VEC. Remarkably, the latter alloy had the lowest VEC value (3.776 compared with 3.896 for the alloy Nb_1.7_Si_2.4_Ti_2.4_Al_3_Hf_0.5_) and its Zone A had even lower VEC values (respectively 3.572, 3.488 and 3.539 for the as cast Zone A, and Zone A heat treated at 800 °C and heat treated at 1200 °C). The alloy design methodology NICE predicts that for good oxidation at 800 and 1200 °C the VEC value of the alloy should be low [4].

### 5.3. Oxidation

The development of oxidation resistance in an alloy requires the presence of an alloying addition that oxidises selectively to produce a protective oxide. The latter requires the oxide to be more stable than that of the base metal. Therefore, a necessary but not sufficient requirement for the formation of a protective oxide on the surface on an alloy is that the oxide is more stable than all possible oxides. Al_2_O_3_ and SiO_2_ are highly stable oxides owing to their low standard free energies of formation and are desirable for oxidation protection at *T* > 1000 °C. In terms of the standard free energy of oxide formation, the oxides of Nb and Ti are nearly as stable as Al_2_O_3_ and SiO_2_. Which oxide is stable on an alloy also depends on metal activities. Changes in the activities of the elements of an alloy can change the composition of oxidation products significantly.

For the oxide of an element to form, the latter must be available at the oxide/alloy or alloy/gas interface and the partial pressure of oxygen in contact with the alloy must exceed the equilibrium pressure for the oxidising reaction at that value of the activity of the element. The Si/SiO_2_ equilibrium pressure is several orders of magnitude higher than that of Al/Al_2_O_3_ and Ti/TiO_2_, both of which are similar [45]. Knowledge of the activity variation within the alloy system in which intermetallic phases exist is necessary to predict correctly the oxide stabilities.

There was internal oxidation in the alloy Nb_1.7_Si_2.4_Ti_2.4_Al_3_Hf_0.5_ but not in the alloy Nb_1.3_Si_2.4_Ti_2.4_Al_3.5_Hf_0.4_. According to Wagner, the critical solute concentration for the transition from internal to external oxidation increases with the solubility and diffusivity of oxygen and decreases with an increase in the solute diffusivity in the alloy [46]. When the oxide of the base metal can form and grow until the more stable oxide of the solute becomes stable and stops the growth of the transition oxide an excess solute above that calculated by Wagner is required [47]. The excess solute concentration increases as the growth rate of the transient oxide increases. In intermetallics the rate of transient oxidation is a more significant factor in determining whether or not protective scale develops. Simulated transport kinetics of oxygen (oxygen penetration depths) in pure Nb and two Nb–Al and Nb–Al–Hf alloys showed significantly reduced oxygen penetration in the ternary alloy [48].

Wagner also showed that the concentration of solute that is required to maintain the growth of an external scale depends on the thermodynamic and diffusional properties of the alloy immediately beneath the oxide [49]. In the case of intermetallic compounds with narrow or no solubility ranges the consumption of the element that forms the external oxide results in the formation of the next intermetallic compound with a lower concentration of the consumed element next to the external oxide. The properties of the lower intermetallic compound determine the ability of the intermetallic to maintain the growth of the protective oxide. In the case of NbAl_3_ the lower compound is Nb_2_Al, which has poor oxidation (see Section 2). In the case of Ti_5_Si_3_ the lower compound is Ti_3_Si which has inferior oxidation behaviour. In the case of TiAl_3_ the lower compounds are Ti_2_Al_5_ and TiAl_2_. In the case of alumina forming Al rich TiAl with α_Al_/α_Ti_ > 1 the lower compound is titania forming Al poor TiAl with α_Al_/α_Ti_ < 1 (α*_i_* is the activity of element *i* = Al,Ti).

Some alloys can form protective oxide at low temperatures and others at high temperatures. The effect of temperature and alloying additions on the selective oxidation of an element is linked with how temperature and alloying element affect oxygen permeability and solute diffusivity in the alloy and the growth rate of transient oxide. Such data is not available for the Nb–Si–Ti–Al–Hf system.

The two alloys of this study do not have “a base metal”. The same is true for the intermetallic compounds in their microstructures, with the exception of TiAl_3_ in the cast alloy Nb_1.7_Si_2.4_Ti_2.4_Al_3_Hf_0.5_ (Section 4.1) and the Al rich TiAl (55 at.% Al) in the alloy Nb_1.3_Si_2.4_Ti_2.4_Al_3.5_Hf_0.4_ that was heat treated at 1200 °C (Section 4.2). Both these compounds were very poor in Nb. In other words, it is not easy to indicate which would be the “lower compound” of the majority of the intermetallics in the microstructures of the two alloys as they oxidised. In addition, the solidification, solute partitioning and growth processes associated with the 5-3 silicide in the alloy Nb_1.3_Si_2.4_Ti_2.4_Al_3.5_Hf_0.4_ resulted in a composite structure (see Section 5.2.2) where the 5-3 silicide core was surrounded by higher not lower compounds.

Do the oxidation responses of the two alloys at 800 °C, where they did not pest, and at 1200 °C, where the alloy Nb_1.3_Si_2.4_Ti_2.4_Al_3.5_Hf_0.4_ formed a continuous thin well adhering αAl_2_O_3_ scale with no internal oxidation, point to some form of “cocktail effect” [50] and therefore unexpected synergies between elements and/or intermetallic phases in each alloy? Were the solubility and diffusivity of oxygen and the solute diffusivities in the alloys affected by synergies between elements and/or intermetallic phases? Which (if any) were the synergistic mixtures of elements and/or phases in each alloy at 800 °C? Why there were no synergies that resulted in exceptional oxidation for the alloy Nb_1.7_Si_2.4_Ti_2.4_Al_3_Hf_0.5_ at 1200 °C? Which were the synergistic mixtures of elements and/or intermetallic compounds that gave the exceptional oxidation of the alloy Nb_1.3_Si_2.4_Ti_2.4_Al_3.5_Hf_0.4_ at 1200 °C? Was the oxidation behaviour of the latter alloy greater than the sum of constituent parts? Was the oxidation at each temperature determined only by activities and partial pressures of oxygen? Was the oxidation of each alloy some combination of the above? We are not able to provide answers to these questions. We shall discuss the oxidation of the two alloys by referring to their starting and/or heat treated microstructures, current knowledge about the oxidation of binary or ternary intermetallic phases and data about the thermal expansion of compounds and oxides.

The starting microstructure of the oxidation specimens of the alloy Nb_1.7_Si_2.4_Ti_2.4_Al_3_Hf_0.5_ consisted of hexagonal and tetragonal Nb_5_Si_3_, TiAl_3_ and (Ti,Nb)Al_3_, TiAl, Ti_2_Al_5_ and TM_2.35_Al_1.65_Si and TM_3.7_Al_3_Si. Regarding the aluminides, only the tri-aluminide was present in the bottom and only the TiAl in the bulk and top of the cast alloy and the Ti_2_Al_5_ was found only in the bulk of the button. After the heat treatment the TM_2.35_Al_1.65_Si and TM_3.7_Al_3_Si compounds were not stable and the tri-aluminide had Nb/Ti > 1 (i.e., it was Nb rich). The fully intermetallic microstructure of this alloy was not free of micro cracks.

The aluminide (Ti,Nb)Al_3_ with Nb/Ti ≈ 1 and the Ti_5_Si_4_, TiSi and hexagonal γNb_5_Si_3_ silicides were present in all parts of the starting microstructure of the oxidation specimens of the alloy Nb_1.3_Si_2.4_Ti_2.4_Al_3.5_Hf_0.4_. The same phases were present after the heat treatments at 800 and 1200 °C and Nb poor TiAl at the latter temperature. Severe cracking of Nb_5_Si_3_ was observed in the microstructure of Nb_1.3_Si_2.4_Ti_2.4_Al_3.5_Hf_0.4_ particularly after the heat treatment at 800 °C. The vol.% of TMAl_3_ was very high in Zone A compared with Zone B and the bulk and top of the button of this alloy.

The main difference between the starting microstructures of the two alloys for the isothermal oxidation experiments were (i) the absence of TiAl and the presence of Ti_5_Si_4_ and TiSi everywhere in the microstructure of the alloy Nb_1.3_Si_2.4_Ti_2.4_Al_3.5_Hf_0.4_; (ii) the presence only of hexagonal 5-3 silicide in the microstructure of the latter alloy and (iii) the significantly higher vol.% of TMAl_3_ in Zone A of this alloy compared with the bulk and top and the low vol.% of TMAl_3_ formed in the bottom of the alloy Nb_1.7_Si_2.4_Ti_2.4_Al_3_Hf_0.5_. Furthermore, in the alloy Nb_1.3_Si_2.4_Ti_2.4_Al_3.5_Hf_0.4_ (a) the TiAl was scarcely present in the bulk and top of the button after the two heat treatments and no TiAl was observed at 800 °C, but at 1200 °C the TiAl in Zone A was very poor in Nb (Ti/Nb = 13); (b) the Ti_5_Si_4_ became richer in Ti and poorer in Nb at 1200 °C compared with 800 °C and (c) at 1200 °C the TiSi had grown significantly compared with the cast microstructure. In both alloys no HfO_2_ particles were observed in the as cast and heat treated conditions and after oxidation at 800 °C. In the alloy Nb_1.7_Si_2.4_Ti_2.4_Al_3_Hf_0.5_ probably there was some hafnia in the scale formed at 1200 °C (see BSE image and Hf and O maps in Figure 11). No hafnia was observed in or below the scale formed on the alloy Nb_1.3_Si_2.4_Ti_2.4_Al_3.5_Hf_0.4_.

Both alloys did not pest at 800 °C and both formed thin scales at this temperature. Remarkably, the alloy Nb_1.3_Si_2.4_Ti_2.4_Al_3.5_Hf_0.4_ did not pest even though its heat treatment at 800 °C indicated a heavily cracked microstructure (Figure 7a). According to the XRD data, at 800 °C the scales of the alloys Nb_1.7_Si_2.4_Ti_2.4_Al_3_Hf_0.5_ and Nb_1.3_Si_2.4_Ti_2.4_Al_3.5_Hf_0.4_ consisted of the same phases, namely Ti niobate(s), Nb, Ti, Si, Hf oxides and TiAl_2_O_5_ but the EDS data indicated “islands” of alumina in the latter, and Si containing mixed oxides in both alloys. In the alloy Nb_1.7_Si_2.4_Ti_2.4_Al_3_Hf_0.5_ there was tetragonal Nb_5_Si_3_, which is known to pest, and Nb rich tri-aluminide which also pests (see Section 2). The suppression of pest in this alloy could be attributed to the low vol.% of the aforementioned two phases. The presence of Ti in the oxide(s) in the scale of this alloy was attributed to the oxidation of Al poor TiAl and Nb rich tri-aluminide. The suppression of pest oxidation in the alloy Nb_1.3_Si_2.4_Ti_2.4_Al_3.5_Hf_0.4_ was attributed to the presence only of non-pesting intermetallic phases in its microstructure. The presence of alumina in the scale formed on this alloy was attributed to the high vol.% of tri-aluminide.

At 1200 °C the scale formed on the alloy Nb_1.7_Si_2.4_Ti_2.4_Al_3_Hf_0.5_ was thicker than that formed on the alloy Nb_1.3_Si_2.4_Ti_2.4_Al_3.5_Hf_0.4_ and exhibited a “layered” structure, (Figure 9c and Figure 11), compared with the thin continuous scale that formed on the latter alloy (Figure 12c). The scale formed on the alloy Nb_1.7_Si_2.4_Ti_2.4_Al_3_Hf_0.5_ consisted of Ti-rich mixed oxide and Al and Ti-rich mixed oxide at the top, beneath it formed a Nb and Si-rich mixed oxide, beneath this was Al and Ti-rich mixed oxide and beneath the latter was a continuous mixture of Al_2_O_3_ (major phase) with dispersed (most likely) Ti-rich oxide. There was also internal oxidation with Al_2_O_3_ forming at interfaces between Nb_5_Si_3_ and TMAl. This oxidation behaviour was attributed to the oxidation of alloyed 5-3 silicides and TiAl forming first transient outer mixed oxides that were either Ti-rich or Ti and Al-rich, followed with inner Nb and Si-rich mixed oxides from the oxidation of silicides and then beneath them Al and Ti rich oxides from the oxidation of aluminides and alloyed 5-3 silicides and then alumina mixed with titania from the oxidation of aluminides. The internal oxidation was attributed to the oxidation of TMAl and alloyed 5-3 silicide (see Section 2).

The scale formed on the alloy Nb_1.3_Si_2.4_Ti_2.4_Al_3.5_Hf_0.4_ at 1200 °C consisted of a continuous αAl_2_O_3_ layer that was about 5 μm thick (Figure 13c and Figure 14). Its formation was attributed to the oxidation of Al rich tri-aluminide, Nb poor and Al rich TiAl. The Ti_5_Si_4_ and TiSi that surrounded the hexagonal 5-3 silicide suppressed the formation of the lower Ti_3_Si silicide and the formation of continuous Ti-rich oxide. The alloying of hexagonal 5-3 silicide with Al suppressed the formation of SiO_2_ beneath the alumina scale (see Section 2).

There was a small weight loss of the alloy Nb_1.7_Si_2.4_Ti_2.4_Al_3_Hf_0.5_ after about 18 h at 800 °C. The weight loss “levelled off” after about 70 h, then there was a small weight increase from 83 to 90 h and then again a small weight loss. Study of the isothermal oxidation of the alloy at 800 °C using thermo-gravimetric analysis coupled with mass spectrometry detected very low signals for species with atomic mass 27 and 44. The former and the latter could correspond respectively to Al and SiO. The SiO could be attributed to the reaction of Si in silicides with SiO_2_ (at the silicide-oxide interface) that gives gaseous SiO [51]. The vapour pressures of SiO at 800 °C calculated from extrapolation of the data of Kubaschewski and Chart [51] and Ferguson and Nuth [52,53] respectively are 9.5 × 10^−4^ Pa and in the range 1.8 × 10^−4^ to 7.8 × 10^−4^ Pa. The Al could be attributed to Al loss from Al rich tri-aluminides. The NbO_2_, TiO and TiO_2_ oxides have significant vapour pressures at significantly higher temperatures, which have been determined for the ranges 1739–1882 °C, 2027–2227 °C and 2027–2227 °C, respectively [54]. Extrapolation to 800 °C gives the vapour pressures of both Ti oxides approximately equal to 1.3 × 10^−12^ Pa, compared with 1.3 × 10^−13^ Pa for NbO_2_.

The weight gain versus time data of the alloy Nb_1.3_Si_2.4_Ti_2.4_Al_3.5_Hf_0.4_ exhibited discontinuities (sharp changes in weight), that were more severe at 800 °C than at 1200 °C (Figure 12c,d) and more severe than those of the alloy Nb_1.7_Si_2.4_Ti_2.4_Al_3_Hf_0.5_ at 800 °C. These were attributed to high stresses that built up in the scale and caused it to crack, thus exposing the substrate to the oxidising atmosphere (see below). Remarkably, even after the severe damage of the scales at 800 °C and 1200 °C this alloy did not pest, the scale was able to “repair” itself and formed alumina at both temperatures. The alumina scale was continuous and had uniform thickness at the higher oxidation temperature.

Oxide scales can be subjected to thermal, compositional and intrinsic stresses. Thermal stresses are caused by differences between coefficients of thermal expansion (CTEs) and compositional stresses can be important in non-stoichiometric oxides. Phase transformations, grain growth, coalescence of oxide islands, and changes in point defect concentrations (i.e., phenomena that induce dimensional changes) can build up intrinsic stresses during the growth of the scale.

The α-cristobalite is tetragonal with lattice constants close to the cubic form and is stable below 200 to 270 °C. The β-cristobalite is cubic and is stable above 200 to 270 °C [55]. The transition temperature depends on composition, defects and strains [56]. On heating, the reconstructive α → β transformation is accompanied by an average volumetric change of 2.8%. On cooling, the displacive (martensitic) β → α transformation [55] is accompanied by a approx. 5% reduction in volume, which causes the crystals to crack [56]. There is an overall expansion of about 0.8% from the temperature of the α → β transformation up to 1027 °C [57]. Both α and β cristobalite are auxetic (i.e., have negative Poisson’s ratio) [55]. In cristobalite, Si can be substituted by Al, the substitution affects the stability of both the α and β cristobalite and the α ↔ β transitions. Al^3+^ occupies Si tetrahedral sites [56]. Up to 2.4 mol% of Al_2_O_3_ can substitute for SiO_2_ in cristobalite [56].

In the oxidation of Ti the main product is TiO_2_ (rutile) and oxides like TiO, Ti_2_O_3_. The Ti*_x_*O_2*x*−1_ Magnelli phases oxidise rapidly to TiO_2_. TiO_2_ can also form as anatase. Anatase I transforms to anatase II (both tetragonal) at 642 °C with no volume change, anatase II transforms to rutile (both tetragonal) at 915 °C with negative volume change. Anatase III transforms to rutile (both tetragonal) at 1150 °C with negative volume change and rutile (tetragonal) transforms to brookite (orthorhombic) at 1300 °C with positive volume change [58]. Niobium can form the NbO, NbO_2_ and Nb_2_O_5_ oxides. TiO and NbO have cubic NaCl structure and large number of vacant sites in both the anionic and cationic sub-lattices. The vacancies in NbO are part of the structure and are very different in character from the random vacancies in TiO. The latter has a wide composition range but the composition range of NbO is very small (≈0.1 at.%) [59]. Rutile has a tetragonal crystal structure and two coefficients α_c_ and α_a_ are needed to represent the expansion of its crystals. Volume expansivity is given by 2α_a_ + α_c_ [60]. Micro-cracking of rutile depends on grain size [61] and the likelihood of micro-cracking is increased with grain size [62].

The different crystallographic forms of stoichiometric Nb_2_O_5_ all transform irreversibly above 1100 °C to monoclinic H-Nb_2_O_5_. The transformation of orthorhombic to monoclinic Nb_2_O_5_ is accompanied by a positive volume change [58]. The lattice thermal expansion of H–Nb_2_O_5_ is anisotropic, the “a” and “c” lattice parameters increase with increasing temperature whereas the “b” parameter and the angle β do not change [63]. This thermal expansion behaviour has been attributed to large variation in the Nb–O distances in the NbO_6_ octahedra of Nb_2_O_5_ [4]. Anisotropy of the CTEs of the grains in a polycrystalline oxide may create internal stresses large enough to cause micro-cracking. The presence of micro-cracks will affect strength, elastic moduli and CTE. This is known to be the case in Nb_2_O_5_.

Aluminium oxide is known to exist in several structures, e.g., γ, δ, θ, α. The γAl_2_O_3_ is cubic and is stable up to 800 °C [64], or 1000 °C [65] and nano γAl_2_O_3_ up to 700 °C, the δ-Al_2_O_3_ is tetragonal or orthorhombic and forms above 800 °C, the θ-Al_2_O_3_ is monoclinic and forms above 900 [64] or 1050 °C [65] or 1100 °C and the αAl_2_O_3_ (trigonal or hexagonal) forms above 1000 °C [66] or 1100 °C [65]. The γAl_2_O_3_ has low permeability to diffusing atoms and ions [67]. Si diffuses in Al_2_O_3_ at *T* ≥ 1100 °C [67].

Mixtures of TiO_2_ and Nb_2_O_5_ with Al_2_O_3_ can have low CTE values. The Ti_2_Nb_10_O_29_ is orthorhombic, and both TiNb_2_O_7_ and AlNb_11_O_29_ have monoclinic structure. The thermal expansion of Ti_2_Nb_10_O_29_ depends on its microstructure. The Nb_2_O_5_ content in TiNb_2_O_7_ also affects the thermal expansion of the latter. Similarly, the content of TiO_2_ and Nb_2_O_5_ in Ti_2_Nb_10_O_29_ and TiNb_2_O_7_ affects thermal expansion. In Ti_2_Nb_10_O_29_ the Nb cations can be replaced by Al. In the mixed oxide 2Al_2_O_3_–98Nb_2_O_5_ the major phase was AlNb_11_O_29_ [68].

The TiAl_2_O_5_ is isomorphous with orthorhombic pseudo-brookite TiMe_2_O_5_. Compounds of the latter structure are highly anisotropic with extremely small CTE along the “a” direction and very large CTEs along the “c” direction [69]. The aluminium titanate has a very anisotropic CTE. Doping of this structure with Al_2_O_3_ and SiO_2_ affects strength. The TiAl_2_O_5_ decomposes to αAl_2_O_3_ and TiO_2_ (rutile) between 800 and 1280 °C [70].

The microstructures of Nb-silicide based alloys contain phases with anisotropic CTEs. The CTE values of the alloys of this study are not known. Table 2 summarises data about the thermal expansion of oxides, silicides and aluminides that were observed in the alloys of this study. The CTE values of Nb_5_Si_3_, Ti_5_Si_3_, TiAl and Ti_5_Si_3_O_0.4_ are average values and that of TiAl_3_ was calculated. Regarding the TiSi silicide, its CTE increases with temperature up to about 227–327 °C and after this temperature it does not change significantly [71]. Table 2 shows that the overall thermal expansion of Nb_2_O_5_ is small compared with the other oxides and most likely similar to that of TiAl_2_O_5_, which is very highly anisotropic.

Thermodynamics shows that both Ti_5_Si_4_ and TiSi or Ti_5_Si_3_ and Ti_5_Si_4_ can be in equilibrium with SiO_2_, and that the Ti_5_Si_3_ can be in equilibrium with Ti_2_O_3_ and SiO_2_ or with TiO and Ti_2_O_3_. There is some doubt about the Ti_5_Si_3_-Ti_5_Si_4_-SiO_2_ equilibrium [26]. It has been suggested that the ternary oxides Ti_2_O_3_·5SiO_2_ and 3Ti_2_O_3_·2SiO_2_ may exist [45]. Ti rich and Nb and Si rich oxides can be present in the scales formed on Nb–silicide based alloys [31,72].

The CTE values of the intermetallic compounds that were present everywhere in the microstructure of the alloy Nb_1.7_Si_2.4_Ti_2.4_Al_3_Hf_0.5_ were similar or close to those of TiO_2_ and α-cristobalite. At 800 °C the presence of Al in the thin scale formed on this alloy could be attributed to Al substituting Si in cristobalite. At 1200 °C there was evidence of cracks in the thicker scale in the “top layer” that consisted of Ti rich mixed oxides and Al and Ti rich mixed oxides and in the “layer” that consisted of Si and Nb mixed oxides (Figure 9c and Figure 11). These cracks could be attributed to thermal, compositional and intrinsic stresses (see above and Table 2) and could have formed during the growth of the scale and/or during the cooling of the specimen.

In the case of the alloy Nb_1.3_Si_2.4_Ti_2.4_Al_3.5_Hf_0.4_ even though the scale was not stable (see above) alumina was formed at both temperatures. The alumina scale had uniform thickness and was continuous at 1200 °C (Figure 13d) but at 800 °C only islands of alumina were observed (Figure 13c). It is likely that in this alloy the concentration of Al (*C*_Al_) was larger than *C*_ther_^Al^ or even *C*_Al_ ≈ *C*_kin_^Al^. At 800 °C there were severe changes in weight (Figure 12c). The glancing angle XRD (Figure 16a) provided evidence for the presence of the low CTE and highly anisotropic aluminium titanate and the BES imaging indicated islands of alumina, probably γAl_2_O_3_, which has a higher CTE compared with the phases that were present everywhere in the microstructure of the alloy (Table 2). The weight changes that occurred during the isothermal oxidation at 800 °C were attributed to the formation of TiAl_2_O_5_ that has highly anisotropic thermal expansion that causes severe micro-cracking, and the high CTE values of TiAl_3_ and γAl_2_O_3_. At 1200 °C the weight changes were not as severe (Figure 12d). This was attributed to the γAl_2_O_3_ → αAl_2_O_3_ transformation, the transformation of TiAl_2_O_5_ to αAl_2_O_3_ (see above), and the lower CTE of αAl_2_O_3_.

Isothermal oxidation experiments allow one to determine the rate of growth of an oxide scale. This is a measure of the performance of an alloy but it is at best a poor yardstick. Oxidation resistance does not depend only on the rate at which an oxide scale thickens but must also consider the ability of the scale to resist the thermally induced stresses associated with cyclic behaviour. We plan to evaluate the latter for the alloy Nb_1.3_Si_2.4_Ti_2.4_Al_3.5_Hf_0.4_, as well as its CTE and mechanical properties. The microstructures and isothermal oxidation of other alumina scale forming alloys of the same alloy system will be discussed in future publication.

## 6. Conclusions

We studied the intermetallic alloys Nb_1.7_Si_2.4_Ti_2.4_Al_3_Hf_0.5_ and Nb_1.3_Si_2.4_Ti_2.4_Al_3.5_Hf_0.4_ that were designed (i) not to have a solid solution in their microstructures; (ii) not to pest and (iii) to form alumina. Both alloys complied with (i) and (ii) and formed thin scales at 800 °C. At 1200 °C the former alloy suffered from internal oxidation and formed alumina intermixed with Ti rich oxide beneath a thick “layered” scale of mixed oxides that contained Ti and/or Al and/or Si and the latter alloy did not experience internal oxidation and formed a thin continuous well adhering α-Al_2_O_3_ scale that was able to repair itself during oxidation at the same temperature.

There was severe macrosegregation of Si in both alloys, which in Nb_1.3_Si_2.4_Ti_2.4_Al_3.5_Hf_0.4_ was almost double that in Nb_1.7_Si_2.4_Ti_2.4_Al_3_Hf_0.5_. The severe macrosegregation of Si contributed to the formation of a “layered” structure in the alloy Nb_1.3_Si_2.4_Ti_2.4_Al_3.5_Hf_0.4_.

The microstructure of the alloy Nb_1.7_Si_2.4_Ti_2.4_Al_3_Hf_0.5_ consisted of hexagonal and tetragonal Nb_5_Si_3_, TiAl_3_ and (Ti,Nb)Al_3_, TiAl, Ti_2_Al_5_. Different aluminides were present in different parts of the button and the tri-aluminide had Nb/Ti > 1.

The (Ti,Nb)Al_3_ with Nb/Ti ≈ 1 and the Ti_5_Si_4_, TiSi and hexagonal γNb_5_Si_3_ silicides were present in all parts of the cast alloy Nb_1.3_Si_2.4_Ti_2.4_Al_3.5_Hf_0.4_ and at 800 and 1200 °C, and Nb poor TiAl at the latter temperature. The “layered” structure of the cast alloy was retained at 800 and 1200 °C.

Both alloys met the “standard definition” of HEAs. The parameters VEC and δ of both alloys respectively were outside and within the range of VEC and δ values for bcc solid solution plus intermetallic(s) HEAs. The parameter Δχ of the alloy Nb_1.7_Si_2.4_Ti_2.4_Al_3_Hf_0.5_ was within the range for bcc solid solution plus intermetallic(s) HEAs but the Δχ of the alloy Nb_1.3_Si_2.4_Ti_2.4_Al_3.5_Hf_0.4_ was outside the lower range of values for bcc solid solution and intermetallic(s) HEAs.

The alloy Nb_1.3_Si_2.4_Ti_2.4_Al_3.5_Hf_0.4_ exhibited strong correlations between the parameters Δχ, δ and VEC, and also sampled a wider range of values of each parameter compared with the alloy Nb_1.7_Si_2.4_Ti_2.4_Al_3_Hf_0.5_. There was a strong correlation only between the parameters Δχ and δ of the latter alloy that was similar to that of the former alloy, which also had the lowest VEC.

## Figures and Tables

**Table 1:** Comparison of alloy parameters for the macrosegregation o Si in the cast alloys [27]. The arrows indicate “direction” of increase of specific parameter. The parameters were calculated as described in Reference [27].

Alloy	MACSi		*T*_m_		Δ*H*_m_		$\frac{\Delta H_{m}}{T_{m}}$		Δ*H*_m_^sd^		Δ*H*_m_^sp^		*T*_m_^sd^		*T*_m_^sp^		$\frac{T_{m}{}^{\mathrm{sd}}}{T_{m}{}^{\mathrm{sp}}}$	
	(at.%)		(K)		(kJ/mol)		(J/molK)		(kJ/mol)		(kJ/mol)		(K)		(K)			
MG1 *	7.4	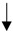	2240	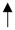	29.1	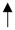	12.99	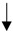	17.93	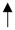	11.17	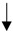	1838.7	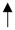	401.3	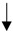	4.58	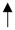
MG2 ^+^	12.8		1747		25.4		14.54		10.8		14.6		1085.3		661.7		1.64	
MG7 **	21.8		1647		24.6		14.97		8.81		15.79		910		737		1.24	

* Nb–18Si–24Ti–5Hf–5Al or alloy NbSiTiHf–5Al in Reference [20]. ^+^ Nb_1.7_Si_2.4_Ti_2.4_Al_3_Hf_0.5_. ** Nb_1.3_Si_2.4_Ti_2.4_Al_3.5_Hf_0.4_.

**Table 2:** Coefficients of thermal expansion, volume thermal expansion, and thermal expansion anisotropy (α_c_/α_a_) of oxides, silicides and aluminides.

Oxide	CTE	T	Volume Thermal Expansion	Thermal Expansion Anisotropy	Ref.
	(×10^−6^ K^−1^)	(°C)	(×10^−6^ K^−1^)	α_c_/α_a_	
γAl_2_O_3_	12.66	27–800	38.87	–	[67]
α-cristobalite	10.3	25	–	–	[55]
TiO_2_	9	27–302	–	–	[73]
	8.4–11.8	–	–	–	[74]
TiO_2_ (rutile)	–	25	23.57	1.28	[75]
	–	50	23.8	1.216	[60]
	–	280	26.78	1.30	[75]
	–	610	31.6	1.473	[60]
TiO_2_ (anatase)		50	15.4	2.05	[60]
		690	39.4	2.147	[60]
αAl_2_O_3_	7.5	1000–1600	–	–	[76]
	–	–	–	1.125	[67]
TiO	6.6	≤477	–	–	[59]
NbO	4.8	≤850	–	–	[59]
β-cristobalite	3.13	300	–	–	[57]
TiNb_2_O_7_	2.3	–	–	–	[77]
Nb_2_O_5_	2.19	25–1000	11.2	1.12	[63]
	1.66	25–1000	–	–	[63]
	1.59–0.48	25–1000	–	–	[78]
TiAl_2_O_5_	0.8–1.3	25–1000	–	–	[79]
αNb_5_Si_3_	8.75 ^+^*	–	–	1.254 *	[2]
βNb_5_Si_3_	10.79 ^+^*	–	–	1.795 *	[2]
Ti_5_Si_3_	–	–	–	3.056 *	[2]
	8.5	800	–	–	[80]
	10.2 ^+^	25	–	–	[81]
	9.25 ^+^	1000	–	–	[81]
Ti_5_Si_3_O_0.4_	10.47 ^+^	25	–	2.5	[81]
TiAl (Ti_44_Al_56_)	10.87 ^+^	27	32.6	0.934	[82]
TiAl_3_	15	25	–	–	[83]

* average value using data from Reference [2]. ^+^ bulk coefficient of thermal expansion (CTE) calculated as (2α_a_ + α_c_)/3.
